# Aurantii Fructus extract alleviates DSS-induced colitis in mice via regulating NF-κB and Nrf2/HO-1 signaling pathways and modulating intestinal microbiota

**DOI:** 10.3389/fnut.2025.1661040

**Published:** 2025-10-16

**Authors:** JunBao Yu, WenYa Mei, JiaYuan Zhu, ZhiHui Wang, XiaoRong Liu, RiBao Zhou, XiangDan Liu

**Affiliations:** ^1^School of Pharmacy, Hunan University of Chinese Medicine, Changsha, Hunan, China; ^2^Key Research Laboratory of Germplasm Resources and Standardized Planting of Genuine Regional Medicinal Materials Produced in Hunan Province, Changsha, Hunan, China; ^3^Key Laboratory of Modern Research of Traditional Chinese Medicine, Education Department of Hunan Province, Changsha, Hunan, China; ^4^Hunan Traditional Chinese Medicine Piece Standardization and Function Technology Research Center, Changsha, Hunan, China

**Keywords:** Aurantii Fructus, ulcerative colitis, intestinal flora, inflammation, oxidative stress

## Abstract

**Background:**

Ulcerative colitis (UC) is a chronic inflammatory bowel disease characterized by progressive loss of intestinal function, highlighting an urgent need for effective therapeutics. This study aimed to investigate the protective effects of Aurantii Fructus extract (AFE) on dextran sulfate sodium (DSS)-induced UC in mice and its impact on the gut microbiota.

**Methods:**

The chemical components of AFE were identified using high-performance liquid chromatography (HPLC). A murine model of DSS-induced colitis was established, and the therapeutic efficacy of AFE was assessed through the disease activity index (DAI), colon length measurement, and histopathological examination. Inflammatory status and oxidative stress markers were evaluated by enzyme-linked immunosorbent assay (ELISA) and western blotting, while the expression of tight junction proteins was analyzed via immunohistochemistry. Additionally, cecal contents were subjected to 16S rRNA gene sequencing to analyze changes in the intestinal microbiota.

**Results:**

AFE treatment significantly alleviated the severity of colitis, as evidenced by reduced DAI scores, attenuated colon shortening, and improved histopathological damage. It restored the expression of the tight junction protein ZO-1 in the colon. AFE also markedly reduced the levels of pro-inflammatory cytokines (TNF-α, IL-6, and IL-1β) and suppressed the activation of the NF-κB pathway. Concurrently, AFE enhanced antioxidant capacity by increasing glutathione (GSH) levels, decreasing malondialdehyde (MDA), and activating the Nrf2/HO-1 signaling pathway. Furthermore, AFE treatment inhibited the proliferation of pathogenic bacteria and restored the homeostasis of the gut microbiota.

**Conclusion:**

The findings demonstrate that AFE confers a protective effect against DSS-induced UC. The underlying mechanism is associated with the inhibition of the NF-κB pathway, activation of the Nrf2/HO-1 antioxidant pathway, enhancement of the intestinal barrier, and restoration of gut microbiota homeostasis.

## 1 Introduction

UC is a chronic, relapsing inflammatory (1) bowel disease characterized by erosive lesions of the mucosa and submucosa, accompanied by symptoms such as diarrhea, bloody stools, abdominal pain, and weight loss. The incidence and prevalence of inflammatory bowel disease increased between 1990 and 2019 and are expected to increase further by 2030 (1). Currently, aminosalicylic acid, steroids, immunosuppressants, and biologics are used to treat UC (2, 3). However, these drugs have some side effects, including nausea, vomiting, headache, diarrhea, nephrotoxicity, and hypokalemia (4). Therefore, it is urgent to find safer and more effective drugs.

At present, it is generally believed that the onset of UC is affected by multiple factors, such as genetics, immune function, intestinal flora, and diet (5). The hallmark features of UC are significant inflammatory and oxidative stress, which are mainly manifested by dysregulated cytokine secretion, a large increase in oxidative stress products, and a large decrease in antioxidant substances (6). In addition, there is evidence that the intestinal microbiota of UC patients undergoes significant changes, with reduced microbial richness and diversity and a large number of pathogenic bacteria. Therefore, fecal microbiota transplantation is often used to treat UC for the purpose of improving the intestinal microbiota (7). The intestinal mucosa of UC patients is damaged, the expression of tight junction proteins (such as ZO-1 and Claudin-1) and mucins is reduced, and the epithelial permeability is increased, which makes it easier for pathogens to invade, ultimately leading to the onset or recurrence of UC (8). In conclusion, inhibiting inflammation, regulating the immune system, reducing oxidative stress, protecting the intestinal mucosa, inhibiting pathogenic microorganisms, and maintaining intestinal flora homeostasis are crucial in the treatment of UC.

AF is the dried unripe fruit of the *Citrus aurantium* L. and its cultivated varieties (9). It is picked in July when the peel is still green. For a long time, AF has been a popular product that is commonly used to prepare functional beverages, possesses homology of medicine and food characteristic (10). Modern research shows that AF has high nutritional value and is rich in a variety of phytochemicals such as flavonoids, volatile oils, coumarins, and organic acids, which have the effects of regulating gastrointestinal motility, anti-cancer, anti-inflammatory, antioxidant, and anti-depressant (11–15). In clinical practice, AF is often used to treat gastrointestinal diseases such as functional dyspepsia, irritable bowel syndrome, and UC (16, 17). However, the effectiveness of AF in treating UC has not been scientifically evaluated, and its mechanism remains unclear.

In this study, we characterized the chemical composition of AFE by HPLC and evaluated the potential of AFE in treating UC mice. We also revealed the efficacy and possible mechanisms of AFE by evaluating the effects of AFE on colon tissue, inflammatory factors, oxidative stress, intestinal barrier function, and intestinal flora community structure in UC mice.

## 2 Materials and methods

### 2.1 Animal and treatment

Male C57BL/6 mice (18–22 g) were purchased from Beijing Vital River Laboratory Animal Technology Co., Ltd. with animal license number SCXL (Beijing) 2021–0006. The mice were kept under 12-h light/dark cycles at 23–25 °C, 50–70% humidity, and had free access to food and water. All animals received humane care, and the research protocol was approved by the Animal Ethics Committee of the Hunan University of Chinese Medicine (Ethical review No: HNUCM21-2311-21). This study was conducted in accordance with the ARRIVE (Animal Research: Reporting of *In Vivo* Experiments) guidelines.

After 1 week of acclimatization, 36 mice were randomly assigned to 6 experimental groups using a computer-generated random number table. This randomization process was performed by a researcher not involved in the subsequent dosing and outcome assessments to ensure allocation concealment. The control group only received pure water. The rest were subjected to a UC induction protocol involving 3.5% DSS in their drinking water for 7 days. During these 7 days, the mice in the SASP treatment group were subjected to intragastric SASP 200 mg/kg administration every day (18). This study used a human clinical dose of AF as the medium dose of AFE in mice (AF: 10 g/day, 60 kg) (19). The dosage for mice was 1.5 g/kg. Three AFE treatment groups (AFE-L, AFE-M, and AFE-H) were treated with 0.75, 1.5, and 3 g/kg AFE daily. The control group and the model group were given the same amount of 0.5% CMC-Na solution.

### 2.2 Materials and reagents

Reference standards with a purity of >98%, including eriocitrin, neoeriocitrin, narirutin, naringin, hesperidin, neohesperidin, meranzin hydrate, poncirin, naringenin, hesperetin, meranzin, nobiletin, tangeretin, and auraptene, were purchased from Chengdu Pfaid Biotechnology Co., Ltd. (Chengdu, China).

Dextran sulfate sodium (DSS) (cat. no. MB5535) was purchased from Dalian Meilun Biotechnology Co., Ltd. (Dalian, China); Fecal occult blood assay kit (cat. no. BC8270) was purchased from Beijing Solarbio Science & Technology Co., Ltd. (Beijing, China); Sulfasalazine (SASP) was purchased from Shanghai Sine Tianping Pharmaceutical Co., Ltd. (Shanghai, China).

Primary rabbit monoclonal antibodies against p-IκBα (cat. no. bsm-60711R), primary rabbit polyclonal antibodies against IκBα (cat. no. bs-1287R) were purchased from Beijing Bioss Biotechnology Co., Ltd. Primary rabbit monoclonal antibodies against Nrf2 (cat. no. 80593-1-RR), p65 (cat. no. 80979-1-RR), p-p65 (cat. no. 82335-1-RR), primary rabbit polyclonal antibodies against *β*-actin (cat. no. 81115-1-RR), GAPDH (cat. no. 10494-1-AP), *α*-Tubulin (cat. no. 11224-1-AP), HRP-conjugated Affinipure Goat Anti-Rabbit IgG (H + L) (cat. no. SA00001-2) were purchased from Proteintech Group, Inc. (Wuhan, China). Primary rabbit polyclonal antibodies against ZO-1 (cat. no. AF5145) were purchased from Affinity Biosciences Co., Ltd. (Changzhou, China).

### 2.3 Detection of active compounds by HPLC

Standards and samples were dissolved in 75% methanol. We measured the content using our pre-established HPLC method (20). The instrument utilized for this study was a 1,260 high-performance liquid chromatograph manufactured by Agilent Technologies (USA).

An Agilent 5 TC-C18 (2) column (250 × 4.6 mm) was utilized. Acetonitrile and 0.1% phosphoric acid aqueous solution were used as mobile phases A and B, respectively. Gradient elution was performed as follows: 0–18 min, 20–21% A; 18–25 min, 21–35% A; 25–26.5 min, 35–48% A; 26.5–30 min, 48–60% A; 30–33 min, 60–68%A; 33–40 min, 68–90% A; and 40–50 min, 90% A. The detection wavelengths were 284 and 318 nm for 0–30 and 30–60 min, respectively. The column temperature was 30 °C, with a volume flow rate and an injection volume of 1.0 mL/min and 10 μL, respectively.

### 2.4 AF collection and extract preparation

AF was collected from Bailuqiao Town, Hanshou, Changde City, Hunan Province, China in July 2023 and dried after collection. Authentication of AF was carried out by Prof. Dr. Zhou Ribao at the Hunan University of Chinese Medicine. Take AF powder and extract it using 80% ethanol as the solvent at a solid-to-liquid ratio of 1:25. Apply ultrasonication at 800 W power for 15 min at 35 °C. After extraction, dry and store the extract.

### 2.5 Disease activity index score

Weight, stool consistency, and blood in the stool were recorded daily. The disease activity index (DAI) score was calculated according to Yuan’s method (21), which was slightly improved. Weight loss percentages: 0 = no weight loss, 1 = 1–5%, 2 = 5–10%, 3 = 10–15%, 4 = > 15%. Stool consistency: 0 = normal, 1 = formed feces that stick, 2 = semi-formed/soft feces, 3 = slurry stool that does not stick, 4 = diarrhea that sticks to the anus. Blood in stool: 0 = negative, 1 = weak positive, 2 = positive, 3 = strong positive, 4 = visible bleeding. The DAI score is the sum of the three parameters.

### 2.6 Histological analysis, immunohistochemistry (IHC), Periodic acid–Schiff (PAS) staining of colon tissues

The researchers who performed the histological scoring and immunohistochemical analysis were blinded to the group identity of the samples. All samples were coded prior to analysis. The intestinal segments were collected at a distance of 2 cm from the anus and fixed in 4% paraformaldehyde for 24 h. The fixed samples were then embedded in paraffin wax, stained with H&E for histopathological analysis in accordance with previously published literature (22) with some modifications.

The scoring criteria were summarized as follows: Intestinal epithelial cell injury (0: normal; 1: mucosal goblet cell loss; 2: mucosal goblet cells were largely lost; 3: Small area of crypt loss; 4: Large area of crypt loss); The extent of inflammatory infiltration (0:normal; 1: inflammatory infiltration of mucosa; 2: extensive inflammatory infiltration of mucosa; 3: inflammatory infiltration of muscle; 4: inflammatory infiltration of muscle); The transverse structure of the colon (0: intact; 1: mucosa damage; 2: submucosa damage; 3: muscle damage). The histological score is the sum of the three parameters.

For IHC, dewaxed 4 μm sections were prepared. Slices were incubated with primary antibodies (ZO-1, 1:200, 4 °C) and secondary biotin antibodies (at 37 °C, 1–2 h). The immune signals were detected with a DAB kit.

Tissue samples were embedded in paraffin and PAS-stained, then analyzed under the microscope.

### 2.7 Detection of inflammatory factors and antioxidant parameters in colon tissue

Homogenize the colon samples, then centrifuged at 1200 × g for 30 min at 4 °C. Collected the upper portion to measure IL-6 (cat. no. JYM0012Mo), TNF-α (cat. no. JYM0218Mo), IL-1β (cat. no. JYM0531Mo), MDA (cat. no. JYM0345Mo), and GSH (cat. no. JYM0743Mo) using ELISA kits (Wuhan Colorful Gene Biotech Co., Ltd.).

### 2.8 16S rRNA gene sequence analysis

The total DNA was extracted from mouse fecal samples and stored at −80 °C, then its content was quantified and its quality checked. The PCR primers 27F (5′-barcode+AGAGTTTGATCMTGGCTCAG-3′), 1492R (5’-ACCTTGTTACGACTT-3′) were synthesized according to the V1 − V9 region of the bacterial 16S rRNA gene, and the target fragments were amplified by PCR. Then, the PCR products were fluorescently quantified, and the libraries were constructed by mixing the samples according to the amount of data required for each sample. Subsequently, the quality control (QC)-qualified libraries were subjected to circular consensus sequencing using a PacBio SequelIIsequencer. Analyze microbiome biological information using QIIME2 version 2022.11. Decode raw sequence data with the demux plugin, trim primers using the cutadapt plugin, then process sequences with the DADA2 plugin for quality filtering, denoising, assembly, and chimera removal. Using the NCBI database, the ASV feature sequences were compared against reference sequences in the database to obtain the corresponding taxonomic information for each ASV. The distance matrix of each sample at the ASV/OTU level was calculated to measure sample diversity and test significance. On the basis of the above results, corresponding graphs were plotted and analyzed by statistical tests.

### 2.9 Western blot analysis

Tissue was homogenized in RIPA buffer with protease/phosphatase inhibitors. Lysate was harvested via centrifugation at 12,000 rpm for 10 min at 4 °C. Protein content was determined using a BCA kit. The proteins were mixed with a buffer, heated at 100 °C for 5 min, and then 20 μg of protein was separated using 10% SDS–PAGE and transferred to PVDF membranes. After blocking, the membranes were incubated with primary antibodies overnight at 4 °C. Primary antibodies dilution ratios: p65 (1:10000), p-p65 (1:5000), p-IκBα (1:1000), IκBα (1:1000), Nrf2 (1:2000), HO-1 (1:1000), GAPDH (1:10000), *β*-actin (1:5000), α-tubulin (1:6000). The membranes were then incubated with secondary antibodies (1:10000) for 2 h at room temperature. Proteins were visualized using an ECL prime kit and analyzed with a GS-700 imager (Bio-Rad Laboratories, Hercules, CA, USA). Protein band intensities were quantified using ImageJ software (National Institutes of Health, Bethesda, MD, USA; version 1.53 t). The corrected density of the target protein band was normalized to the corrected density of its corresponding loading control band from the same sample lane to account for variations in protein loading and transfer efficiency.

### 2.10 Statistical analysis

Data were expressed as mean ± SD (x̄ ± s). GraphPad Prism software, version 9.5, was used for statistical analysis and plotting. One-way ANOVA followed by Tukey–Kramer multiple comparisons test was used for parametric data, and the Wilcoxon test was used for non-parametric data. *p* < 0.05 and *p* < 0.01 indicate statistical significance and highly significant, respectively.

## 3 Results

### 3.1 HPLC detection of AFE active components

**Figure 1:**
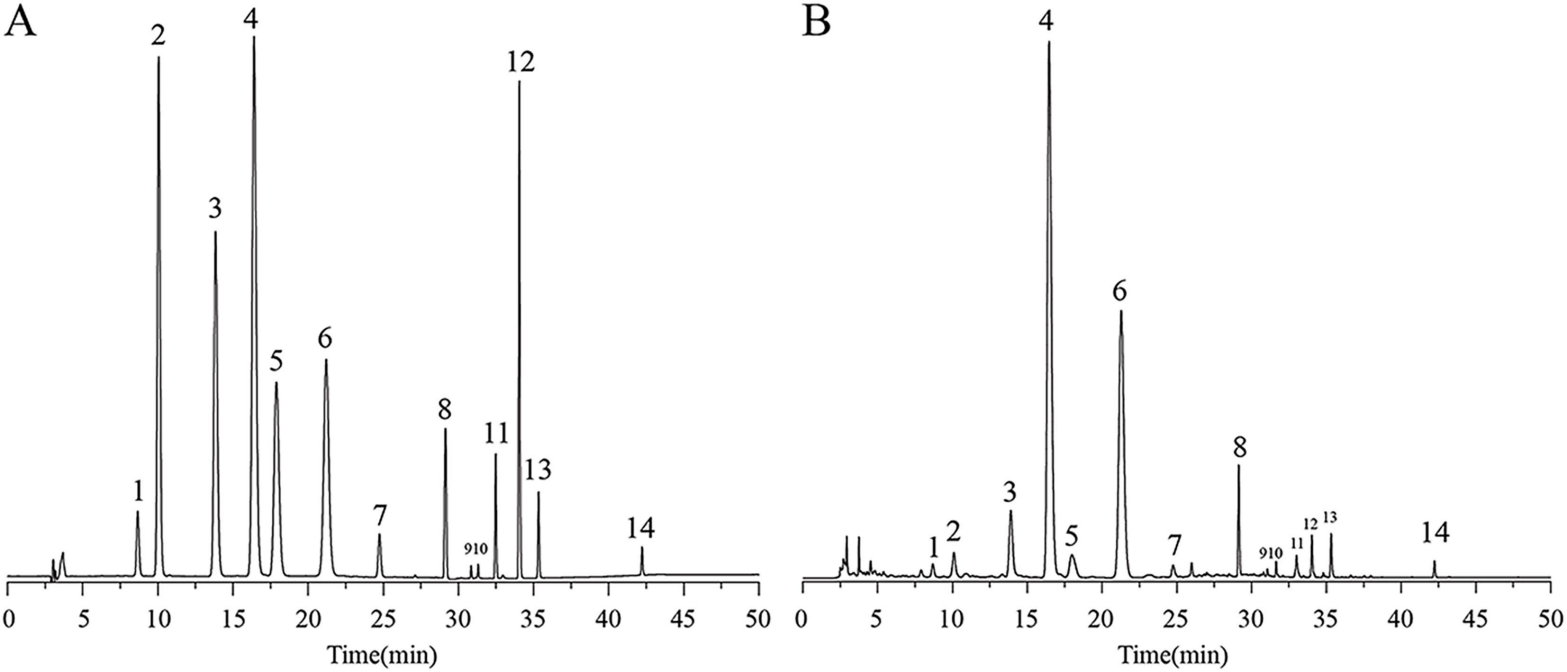
HPLC chromatogram of 14 active ingredients in AFE. **(A)** Mixed standards. **(B)** AFE sample.1: eriocitrin, 2: neoeriocitrin, 3: narirutin, 4: naringin, 5: hesperidin, 6: neohesperidin, 7: meranzin hydrate, 8: poncirin, 9: naringenin, 10: hesperetin, 11: meranzin, 12: nobiletin, 13: tangeretin, 14: auraptene. HPLC, high-performance liquid chromatography.

In this study, the 14 active ingredients in AFE were analyzed using a pre-established HPLC method (Figures 1A,B), and the contents of eight of them (calculated in terms of crude drug amount) were determined, as shown in Table 1. The contents of naringin (88.42 mg/g), neohesperidin (46.06 mg/g), and narirutin (9.25 mg/g) in AFE were relatively high, accounting for 90.8% of the total content of the eight active ingredients, and they are the main active ingredients of AFE. However, these eight active ingredients only account for 15.8% of the total amount of AFE, indicating that there are still a large number of unknown ingredients in AFE.

**Table 1:** Contents of eight active compounds in AFE (calculated in terms of crude drug amount) (mg/g).

Components	Regression equation	Correlation coefficient	Linear range (mg/mL)	Contents (mg/g)
Eriocitrin	*y* = 19,143x−20.206	0.9996	0.006–0.180	1.25
Neoeriocitrin	*y* = 20,604x−31.63	0.9998	0.009–0.280	2.64
Narirutin	*y* = 18,278x−6.9787	1.0000	0.014–0.460	9.25
Naringin	*y* = 17,003x−207.23	0.9995	0.109–3.5	88.42
Hesperidin	*y* = 21,839x−8.9123	0.9999	0.004–0.134	4.02
Neohesperidin	*y* = 19,491x + 97.98	1.0000	0.038–1.2	46.06
Poncirin	*y* = 18,544x + 3.0268	1.0000	0.009–0.300	5.38
Nobiletin	*y* = 33,449x−4.4005	1.0000	0.003–0.100	1.23

### 3.2 AFE alleviates DSS-induced UC in mice

**Figure 2:**
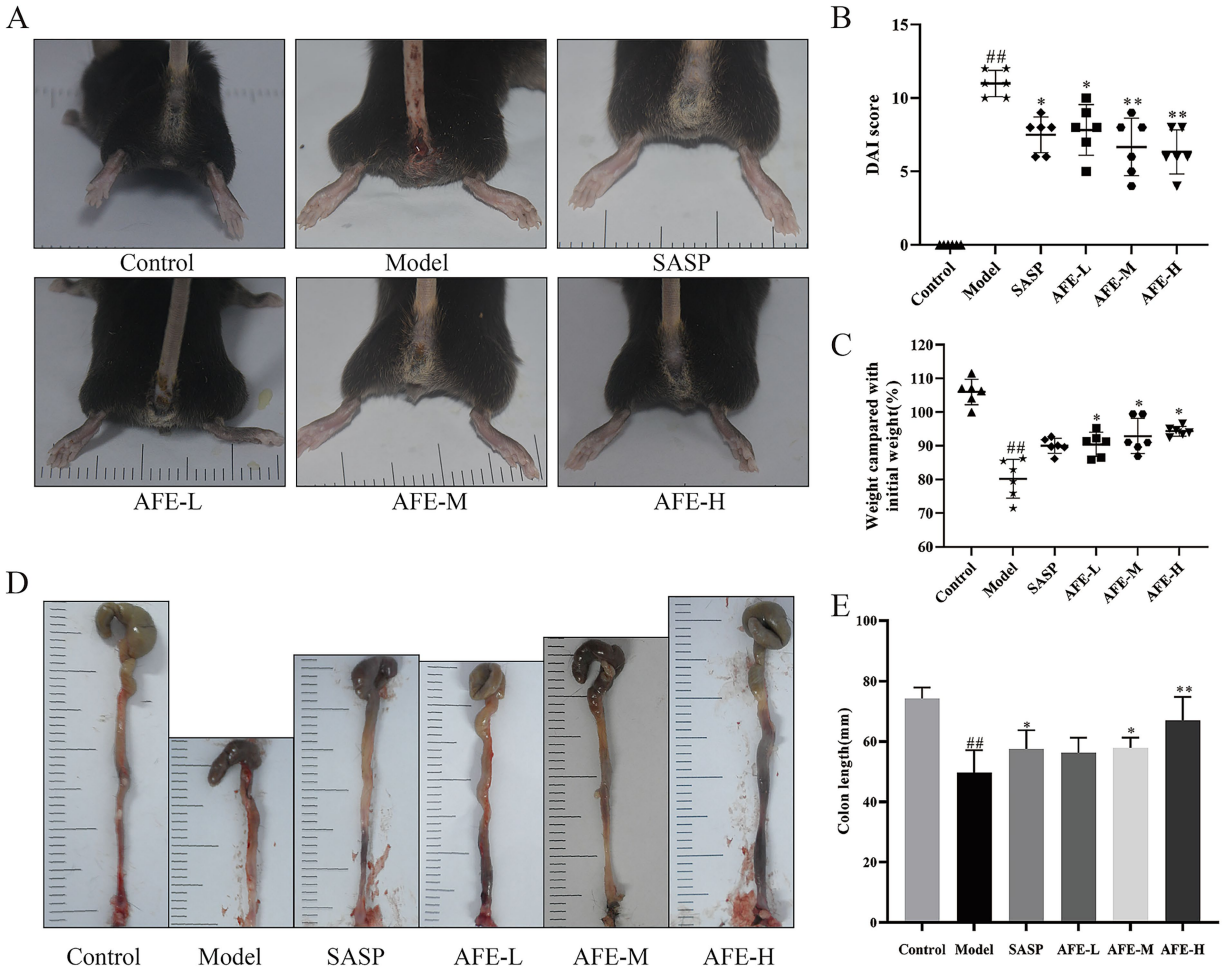
FD intervention relieves DSS-induced UC in mice. Symptoms of hematochezia and diarrhea in mice **(A)**. Weight compared with initial weight **(B)** and Disease activity index **(C)**. The colon length **(D,E)**. x̄ ± s, n = 6. ^##^*p* < 0.01 vs. control group; ^*^p < 0.05, ^**^p < 0.01 vs. model group.

To study the effect of AFE on UC, a UC model was constructed by one-week DSS induction, with modelling and treatment at the same time. After 7 days of modeling, the model group had symptoms of hematochezia and diarrhea (Figure 2A). By day 7, mice in the model group had a significantly higher DAI (11.13 ± 0.64) (Figure 2B, *p* < 0.01), had lost significantly more weight (83.83 ± 6.5%) (Figure 2C, *p* < 0.01) and had significantly shorter colon lengths (49.50 ± 0.64 mm) (Figures 2D,E, *p* < 0.01). Compared with the model group, each dose of AFE reduced the DAI of UC mice (*p* < 0.05; *p* < 0.01), inhibited the weight loss caused by DSS (*p* < 0.05). AFE-M (56.87 ± 5.27 mm) and AFE-H (60.70 ± 8.84 mm) treatments inhibited the colonic shortening caused by DSS (*p* < 0.05; *p* < 0.01), and AFE-L (52.67 ± 2.98 mm) only had a tendency to inhibit. The results showed that AFE could alleviate DSS-induced UC in mice.

### 3.3 Hematoxylin and eosin (H&E) staining and immunohistochemical analysis

**Figure 3:**
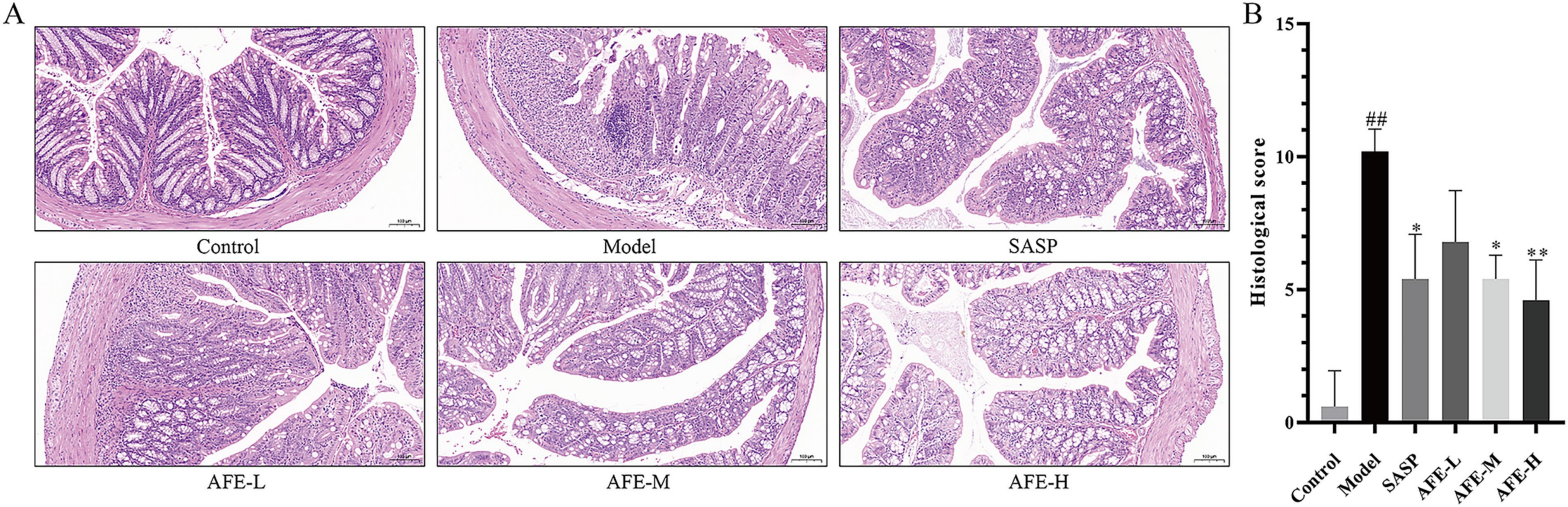
AFE attenuates colonic pathological damage in acute UC mice. **(A)** Representative images showing colon pathologic damages with hematoxylin and eosin (H&E) staining, (200 × magnification). **(B)** Histological scores of colons. x̄ ± s, images are representative of *n* = 5 mice per group. ^##^*p* < 0.01 vs. control group; ^*^p < 0.05, ^**^p < 0.01 vs. model group.

The colon tissue exam results (Figure 3A) showed that the colonic mucosal structure of the model group was seriously damaged, with extensive crypt deficiency and significant inflammatory cell infiltration. Following the administration of AFE, a substantial alleviation of the DSS-induced histological injury was observed. The colon tissues in all treatment groups showed a significant attenuation of damage. Inflammatory cells decreased, goblet cells and epithelial cells recovered, and ulcer surfaces shrank. Compared with the model group, AFE-L group could not significantly reduce the histological score of colons (Figure 3B), indicating that the administration of 0.75 g/kg AFE did not alleviate DSS-induced colon injury. Compared with the AFE-L group, the SASP, AFE-M, and AFE-H groups had better therapeutic effects and were significant compared with the model group (*p* < 0.05, *p* < 0.05*, p* < 0.01; Figure 3B). Inflammatory cells were only seen in the colon mucosa and not much in the submucosa.

### 3.4 AFE protected the intestinal mucosal barrier in mice with DSS-induced UC

**Figure 4:**
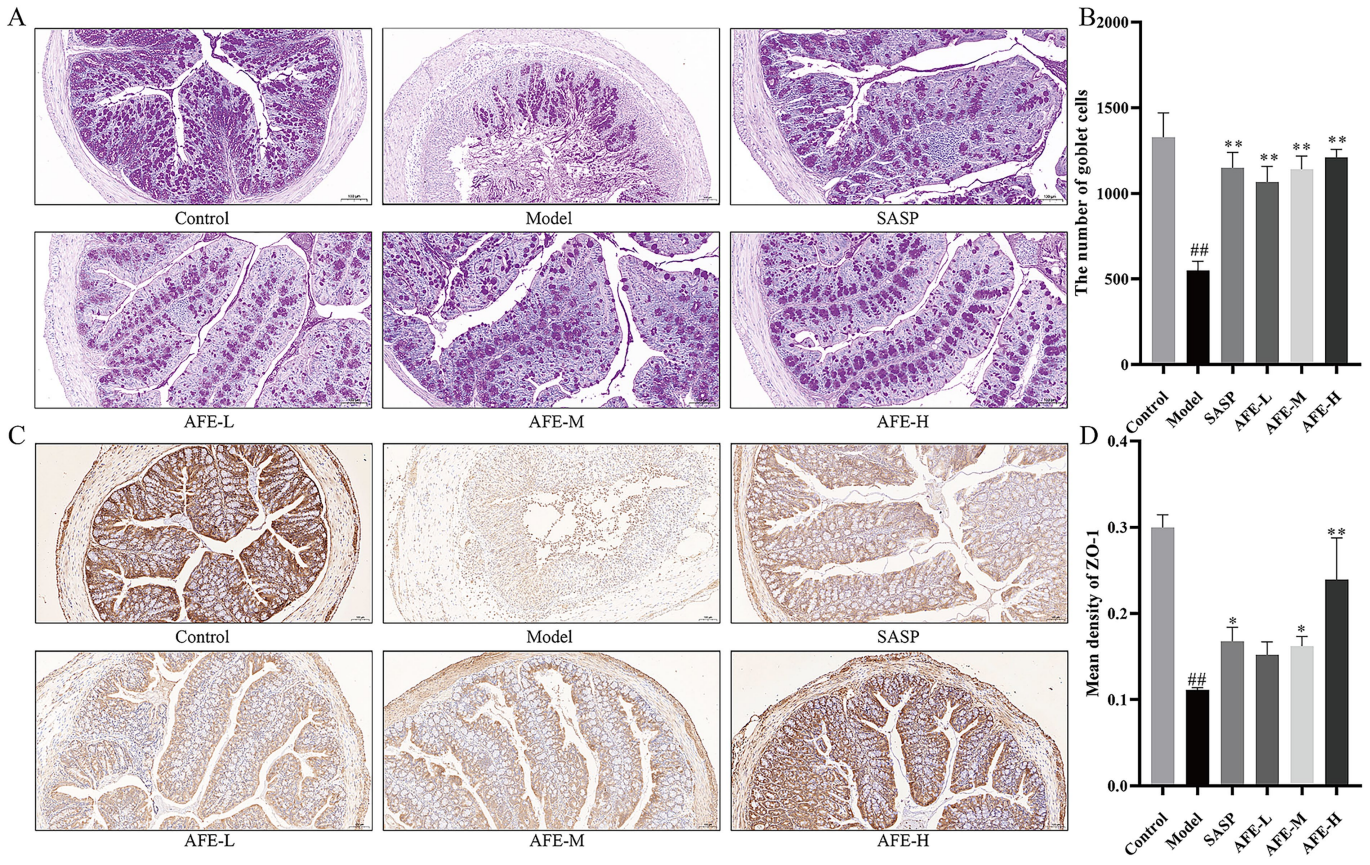
The intestinal mucosal protective effect of AFE. **(A,B)** PAS staining and statistical analysis of the results (200 × magnification). **(C,D)** IHC staining of ZO-1 and statistical analysis of the results (200 × magnification). x̄ ± s, images are representative of *n* = 3 mice per group. ^##^*p* < 0.01 vs. control group; ^*^ *p* < 0.05, ^**^p < 0.01 vs. model group.

Figures 4A,B show the results of the PAS staining. Goblet cells produce mucin, protecting the intestine from bacteria. They were vital for the mucosal barrier. The control group had many purple-red spots, indicating intact goblet cells. Mice in the model group had significantly fewer colon goblet cells than those in the control group (*p* < 0.01). Loss of goblet cells in the colon tissue of mice with UC improved following each dose of AFE (*p* < 0.01), indicating that AFE protected intestinal goblet cells in mice with UC and thereby protected the intestinal mucosal barrier.

The IHC results of ZO-1 in the colon tissues are shown in Figure 4C. ZO-1 was located on the cell membrane. The control group was brown with dark staining and strong ZO-1 expression; the model group was light yellow with light staining and weak ZO-1 expression. Statistical analysis was shown in Figure 4D. Compared with the control group, the average optical density of ZO-1 in the colon tissue of the model group mice was significantly lower (*p* < 0.01). After AFE intervention, the average optical density of ZO-1 in the AFE-L group showed an upward trend compared with the mice in the model group. AFE-M and AFE-H significantly reversed the reduction in ZO-1 protein expression in colon tissue after modelling (*p* < 0.05; *p* < 0.01). These results suggested that AFE can restore the colonic epithelial barrier in DSS-treated mice.

### 3.5 Detection results of inflammatory factors and antioxidant parameters

**Figure 5:**
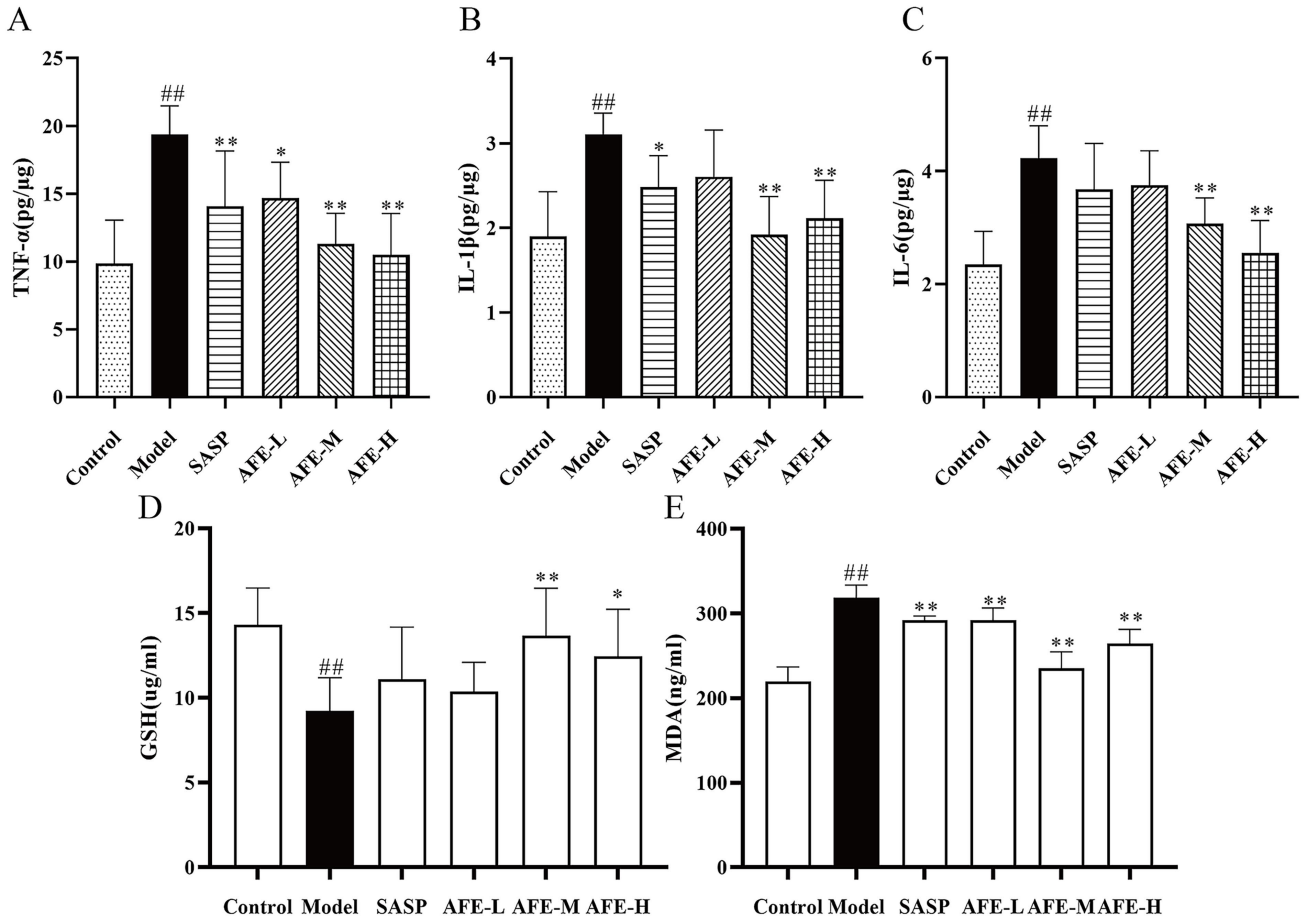
Anti-inflammatory and antioxidant effects of AFE on UC. Treatment with AFE down-regulated plasma TNF-α **(A)**, IL-1β **(B)**, IL-6 **(C)**, MDA **(D)**, and GSH **(E)** levels in colon tissues. x̄ ± s, *n* = 5. ^#^*p* < 0.05, ^##^*p* < 0.01 vs. control group; ^*^p < 0.05, ^**^p < 0.01 vs. model group.

As shown in Figure 5, TNF-α (*p* < 0.01, Figure 5A), IL-1β (*p* < 0.01, Figure 5B), and IL-6 (*p* < 0.01, Figure 5C) levels were increased in the model group, showing DSS can induce inflammation. TNF-α content was significantly reduced in all treatment groups in comparison with the model group (*p* < 0.01). Following oral administration of 0.75 and 1.5 g/kg AFE, secretion of IL-6 and IL-1β was significantly downregulated (*p* < 0.01). In addition, the expression levels of GSH and MDA were measured to further understand the extent of the oxidative stress. Model group recorded a significant decrease in the colon GSH (*p* < 0.01, Figure 5D) contents and typically showed a significant increase in MDA (*p* < 0.01, Figure 5E) level as compared to the control group, indicating the possible formation of oxidative damage. Treatment with AFE at doses of 1.5 g/kg and 3 g/kg significantly increased GSH contents (*p* < 0.01, *p* < 0.05), and significantly decreased MDA level (*p* < 0.01) as compared to the model group.

### 3.6 AFE’S effects on NF-κB pathway inhibition in UC mice

**Figure 6:**
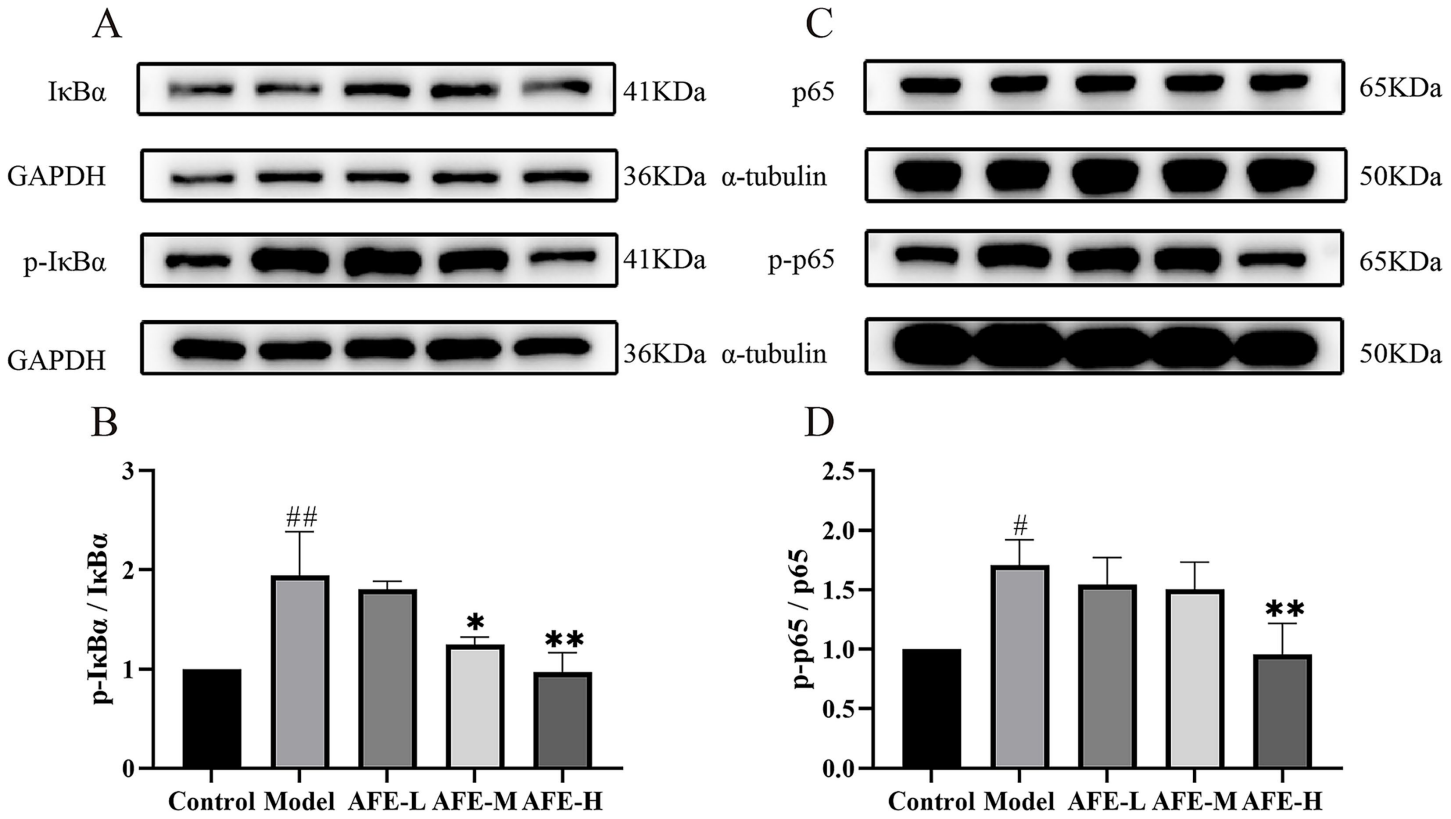
AFE’s effect on the NF-κB pathway in UC mice. AFE inhibits the NF-κB pathway in UC mice. Western blot of protein expression of p-IκBα and IκBα **(A,B)**, p-p65 and p65 **(C,D)** in colon tissue. x̄ ± s, *n* = 3. ^#^*p* < 0.05, ^##^*p* < 0.01 vs. control group; ^*^p < 0.05, ^**^p < 0.01 vs. model group.

The relative expression of p-IκBα (Figures 6A,B) and p-p65 (Figures 6C,D) in colon tissue was significantly increased in the model group compared with the control (*p* < 0.01, *p* < 0.05), while AFE-H treatment significantly reduced the relative expression of p-p65 and p-IκBα (*p* < 0.01). These results might suggest that AFE-H protected DSS-induced experimental UC by inhibiting the NF-κB pathway.

### 3.7 AFE’S effects on Nrf2/HO-1 pathway activation in UC mice

**Figure 7:**
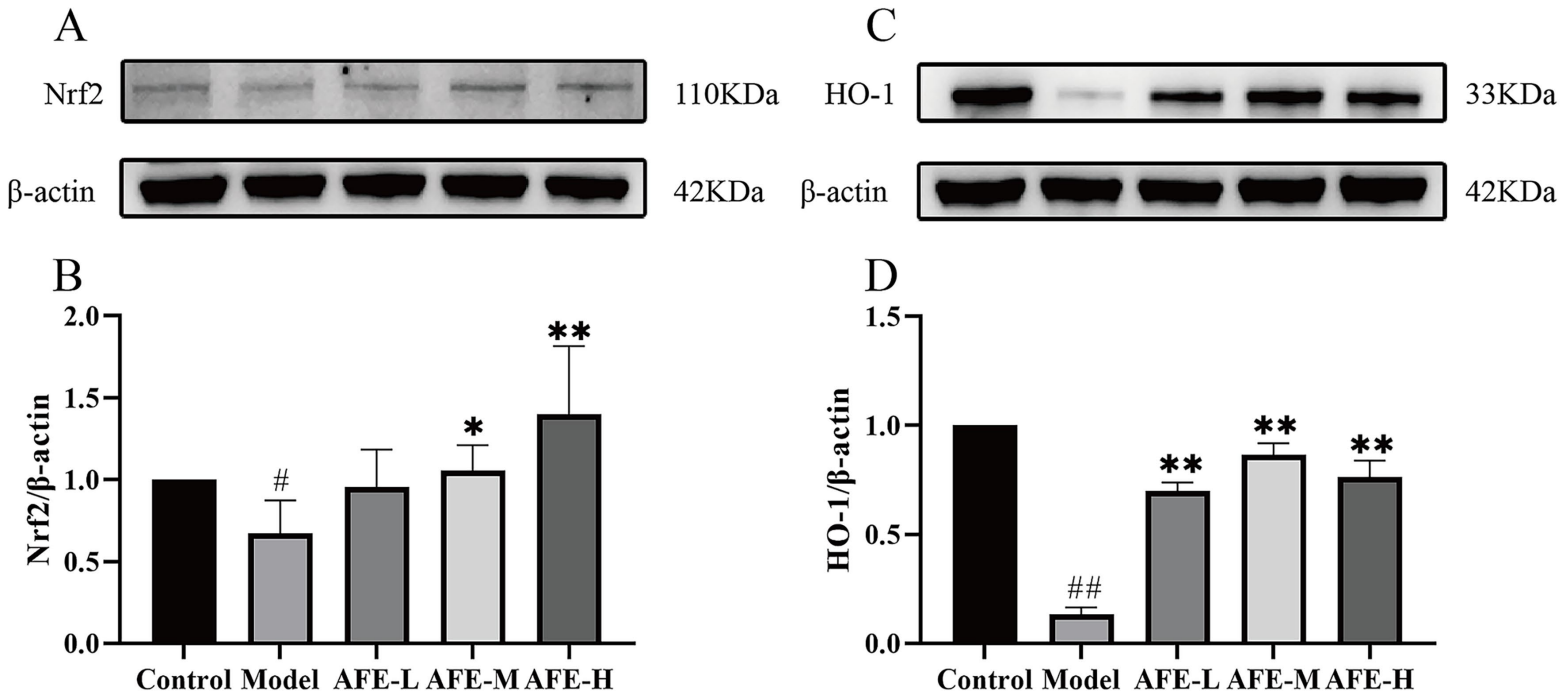
Activation of AFE on the Nrf2/HO-1 signaling pathway in UC mice. Activation of the Nrf2/HO-1 pathway by AFE in UC mice. Western blot analysis of Nrf2 **(A,B)** and HO-1 **(C,D)** protein expression in colon tissue. x̄ ± s, *n* = 3. ^#^*p* < 0.05, ^##^*p* < 0.01 vs. control group; *p < 0.05, **p < 0.01 vs. model group.

Nrf2 (Figures 7A,B) and HO-1 (Figures 7C,D) protein expression was assessed by western blotting. Compared with the control, the model group had significantly lower Nrf2 (*p* < 0.05) and HO-1(*p* < 0.01) levels, showing that the Nrf2/HO-1 pathway was blocked in the colon tissue. Compared with the model, AFE treatment increased Nrf2(*p* < 0.05) and HO-1(*p* < 0.01) expression, suggesting that Nrf2/HO-1 pathway activation may be a therapeutic mechanism.

### 3.8 Effects of AFE fraction on intestinal flora diversity in UC mice

**Figure 8:**
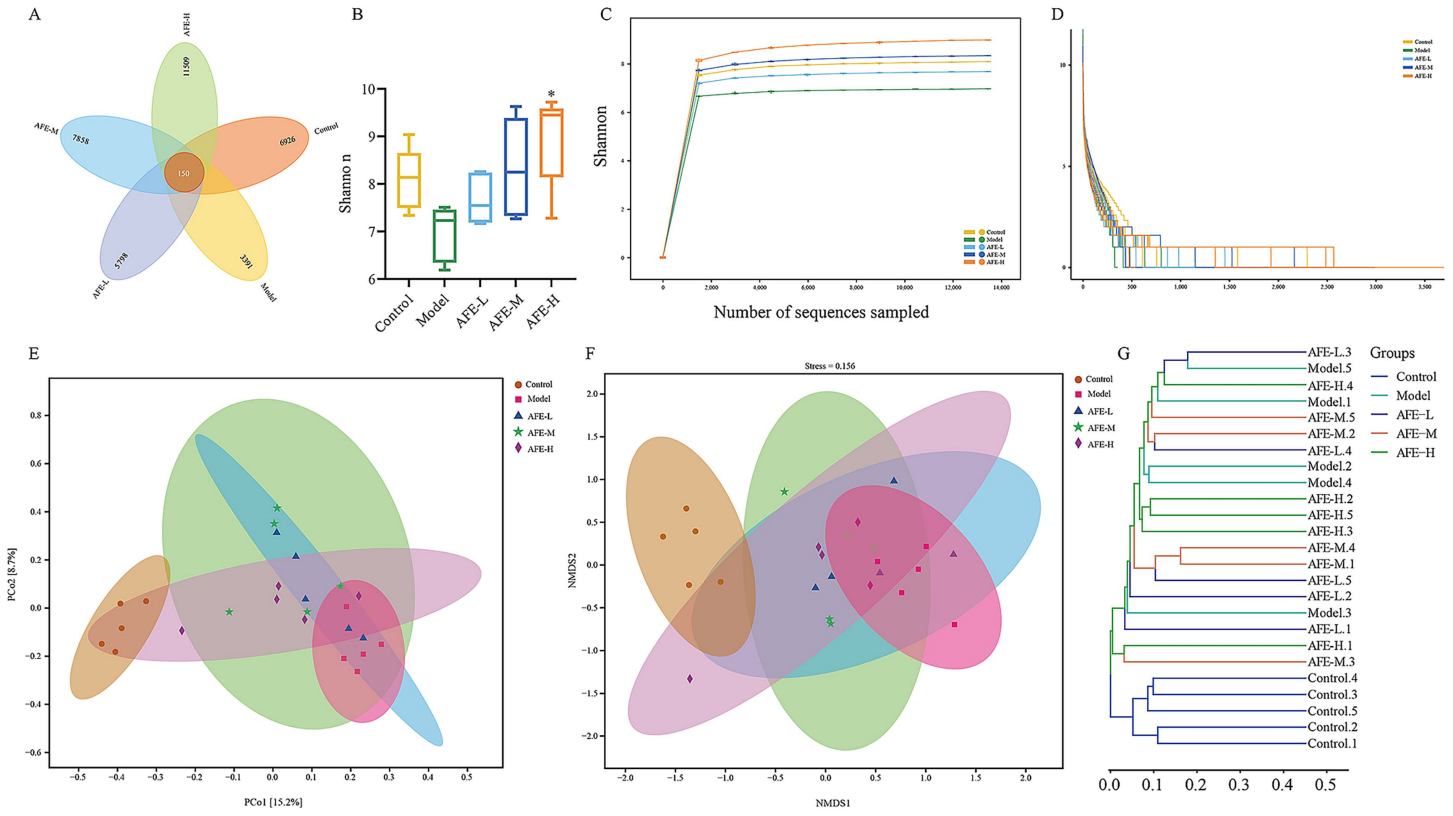
Effects of AFE on intestinal flora diversity in UC mice. Venn diagram **(A)**; Shannon **(B)**; Rarefaction curves **(C)**; Rank abundance curve **(D)**; Principal coordinate analysis (PCoA) plot of Bray-Curtis distance **(E)**; Non-metric multidimensional scaling (NMDS) analysis plot of Bray-Curtis distance **(F)**; Bray-Curtis cluster tree **(G)**. x̄ ± s, *n* = 5. ^*^p < 0.05 vs. model group.

The experiments have shown that the AFE was effective in the treatment of UC. So, we extracted DNA and sequenced the 16S rRNA genes to explore how AFE affects intestinal flora in UC mice. A total of 34,882 OTUs were identified using 97% sequence similarity. A Venn diagram shows the number of OTUs in common and unique to the groups (Figure 8A). These results showed that the 5 groups shared 150 OTUs. The numbers of OTUs were 6,776, 3,241, 5,648, 7,708, and 11,359 for the control, model, AFE-L, AFE-M, and AFE-M groups, showing that the samples were similar and unique at the OTU level.

Alpha diversity was used to assess gut microbiota. The Shannon index decreased in the model group compared to the control group (Figure 8B). Notably, after feeding AFE, the Shannon index was significantly increased in the AFE-H (*p* < 0.05) group and obviously increased in the AFE-L and AFE-M groups compared with the model group. The Shannon index of the OTU level was set as the vertical axis in the rarefaction curve, which tends to flatten, indicating that the sample sequencing volume is sufficient (Figure 8C). The rank-abundance curve explained diversity as species richness and evenness. The curve declined and extended far, indicating high diversity (Figure 8D).

To understand the effects of AFE on the intestinal microflora profile of UC mice, principal coordinate analysis (PCoA) (Figure 8E) and non-metric multidimensional scaling analysis (NMDS) (Figure 8F) of Bray-Curtis distance were performed based on OTU, whose confidence level of the ellipse is 0.95. It can be seen from the PCoA plot that the model group and control group were significantly separated. The AFE treatment groups were different from the model and control groups. The mice’s intestinal flora changed after the model was established. AFE treatment could partly regulate abnormal changes and form a different intestinal flora profile from the control and model groups. NMDS also showed a similar trend to PCoA. As shown in the Bray-Curtis cluster tree diagram (Figure 8G), the model group was far removed from the control group. The AFE treatment groups were closer to the control group (Figure 8F). These results further demonstrated that the intestinal flora composition of UC mice changed, but AFE treatment made these changes closer to the control group’s.

### 3.9 Effects of AFE on intestinal flora structure in UC mice

**Figure 9:**
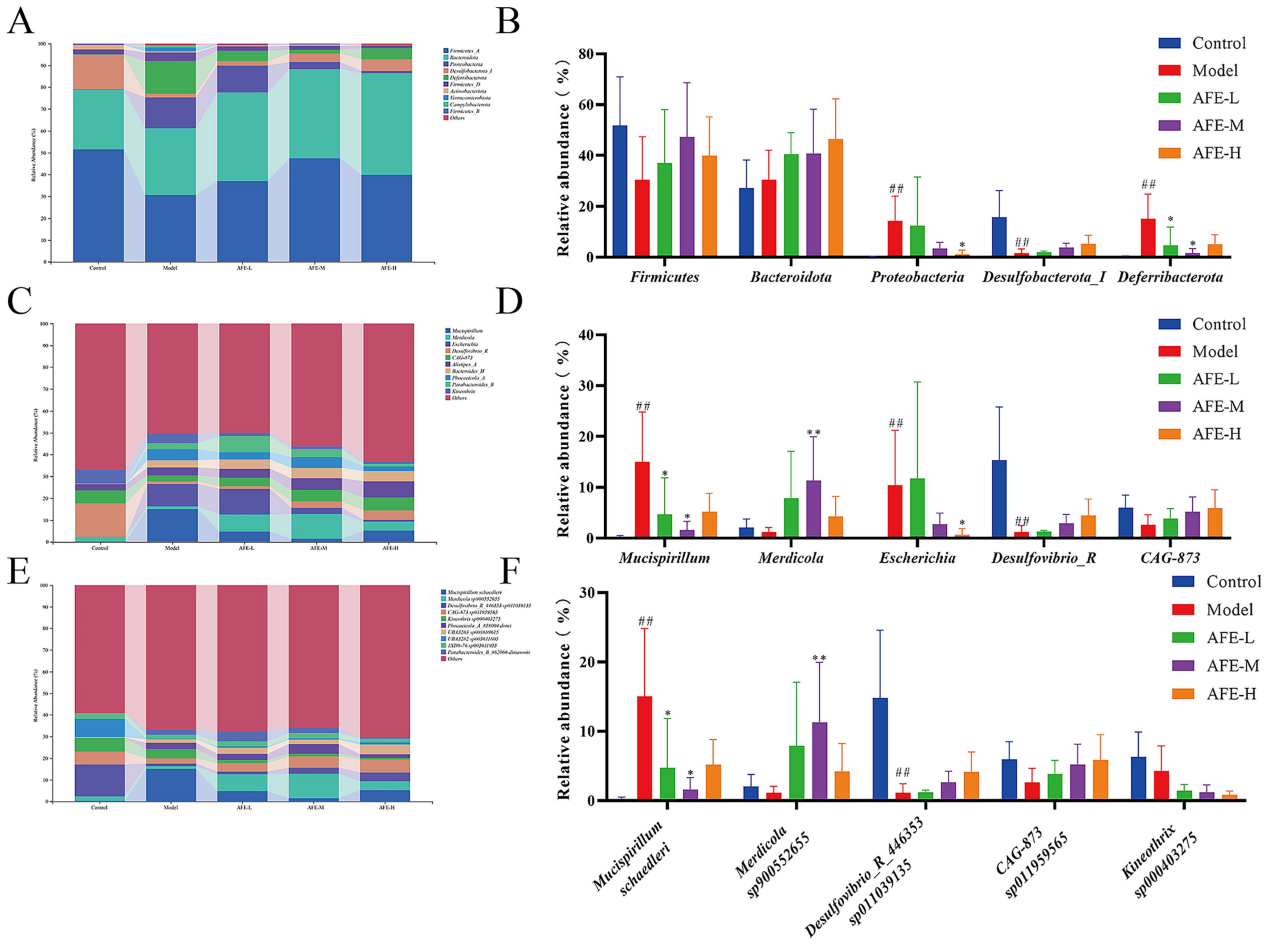
Effects of AFE on intestinal flora structure in UC mice. Difference analysis of intestinal flora at Phylum **(A,B)**, Genus **(C,D)**, Species **(E,F)** levels in mice of each group. x̄ ± s, *n* = 5. ^##^*p* < 0.01 vs. control group; ^*^p < 0.05, ^**^p < 0.01 vs. model group.

At the phylum level (Figures 9A,B), the intestinal microbial community of mice was mainly composed of *Firmicutes*, *Bacteroidota*, *Proteobacteria*, *Desulfobacterota_I*, and *Deferribacterota*. Compared with the control group, the intestinal tract of mice in the model group had a higher abundance of *Proteobacteria* and *Deferribacterota* (*p* < 0.01) and a lower abundance of *Desulfobacterota_I* (*p* < 0.01). Compared to the model group, AFE-H and AFE-L/M significantly reduced *Proteobacteria* and *Deferribacterota* (*p* < 0.05).

At the genus level (Figures 9C,D), the intestinal microbial community composition of mice was dominated by *Mucispirillum*, *Merdicola*, *Escherichia*, *Desulfovibrio_R*, and *CAG-873*. *Mucispirillum* and *Escherichia* levels were higher in the model group than in the control (*p* < 0.01). After treatment with AFE-L and AFE-M, *Mucispirillum* levels dropped (*p* < 0.05). The *Escherichia* was significantly reduced after AFE-H treatment (*p* < 0.05). In addition, compared with the control group, the *Desulfovibrio_R* in the model group was reduced (*p* < 0.01), *Merdicola* and *CAG-873* showed a decreasing trend, and the number of these types increased after treatment.

At the species level, intestinal dominant species were *Mucispirillum schaedleri*, *Merdicola sp900552655*, *Desulfovibrio_R_446353 sp011039135*, *CAG-873 sp011959565* and *Kineothrix sp000403275*. Compared to the control group, the relative abundance of *Mucispirillum schaedleri* was increased in the model group (*p* < 0.01). After treatment with AFE-L and AFE-M, the relative abundance of *Mucispirillum schaedleri* was significantly decreased (*p* < 0.05). Compared with the control group, the relative abundance of *Desulfovibrio_R_446353 sp011039135* was significantly reduced in the model group (*p* < 0.01), *Merdicola sp900552655*, *CAG-873 sp011959565* and *Kineothrix sp000403275* showed a decreasing trend. AFE-M increased the relative abundance of *Merdicola sp900552655* (*p* < 0.05). AFE increased the relative abundance of *Desulfovibrio_R_446353 sp011039135* and *CAG-873 sp011959565*, and decreased the relative abundance of *Kineothrix sp000403275*, but there is no significance (*p* > 0.05). Results indicated that AFE held potential for improving gut microbiota imbalance, increasing microbial diversity, and stabilizing the microbiome in mice with UC.

### 3.10 Effects of AFE on intestinal flora function in UC mice

**Figure 10:**
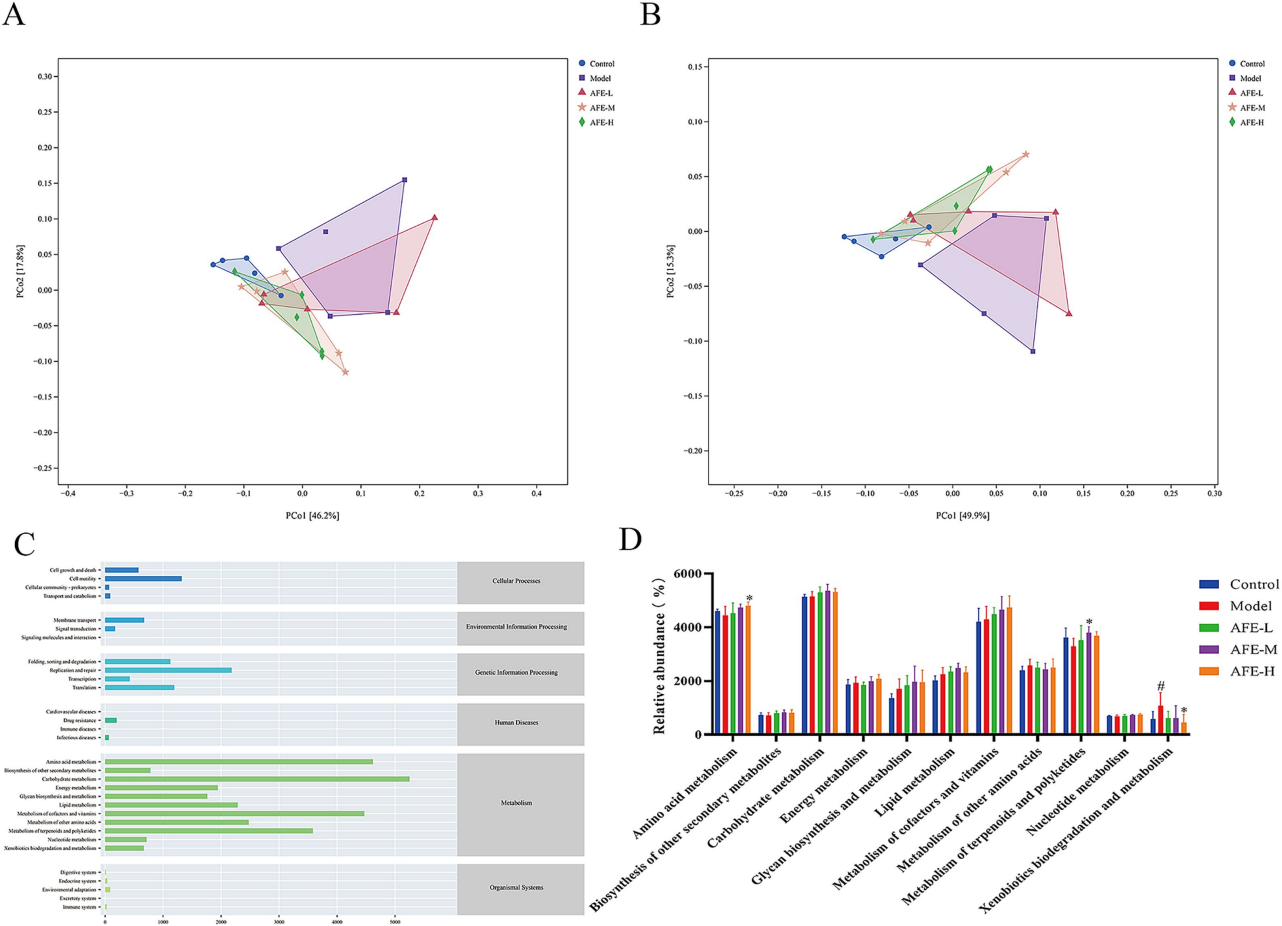
Effects of AFE on intestinal flora function in UC mice. PCoA diagram of KEGG functional units **(A)**; PCoA diagram of MetaCyc functional units **(B)**; Abundance diagram of KEGG functions **(C)**; Functional analysis of metabolic pathways in different treatment groups **(D)**. x̄ ± s, *n* = 5. ^#^*p* < 0.05 vs. control group; *p < 0.05 vs. model group.

To understand changes in intestinal flora function caused by AFE, PCoA analysis was performed based on KEGG and MetaCyc to predict the function of the bacterial flora. The results showed clear separation between the control and model groups (Figures 10A,B). The functions of intestinal flora are generally divided into 6 categories (Figure 9C). The second level includes 31 sub-functional categories, among which metabolism accounts for the largest proportion. Intestinal flora plays a major role in carbohydrate metabolism, amino acid metabolism, and the metabolism of cofactors and vitamins. In Figure 9D, compared with the model group, the amino acid metabolism in the AFE-H group was improved (*p* < 0.05), and the metabolism of terpenoids and polyketides in the AFE-M group was improved (*p* < 0.05), xenobiotics biodegradation and metabolism were significantly reduced in the AFE-H group (*p* < 0.05). This finding was consistent with the fundamental role of the gut microbiota in facilitating metabolic processes. The enrichment of these pathways suggested that AFE components act as substrates or regulators for gut microbes. Studies have shown that natural flavonoids from plants may boost beneficial metabolites like short-chain fatty acids (SCFAs), which could help with UC (23). Therefore, the beneficial effect of AFE in UC was associated with changes in the metabolic activity of the gut microbiota.

### 3.11 Correlation analysis

**Figure 11:**
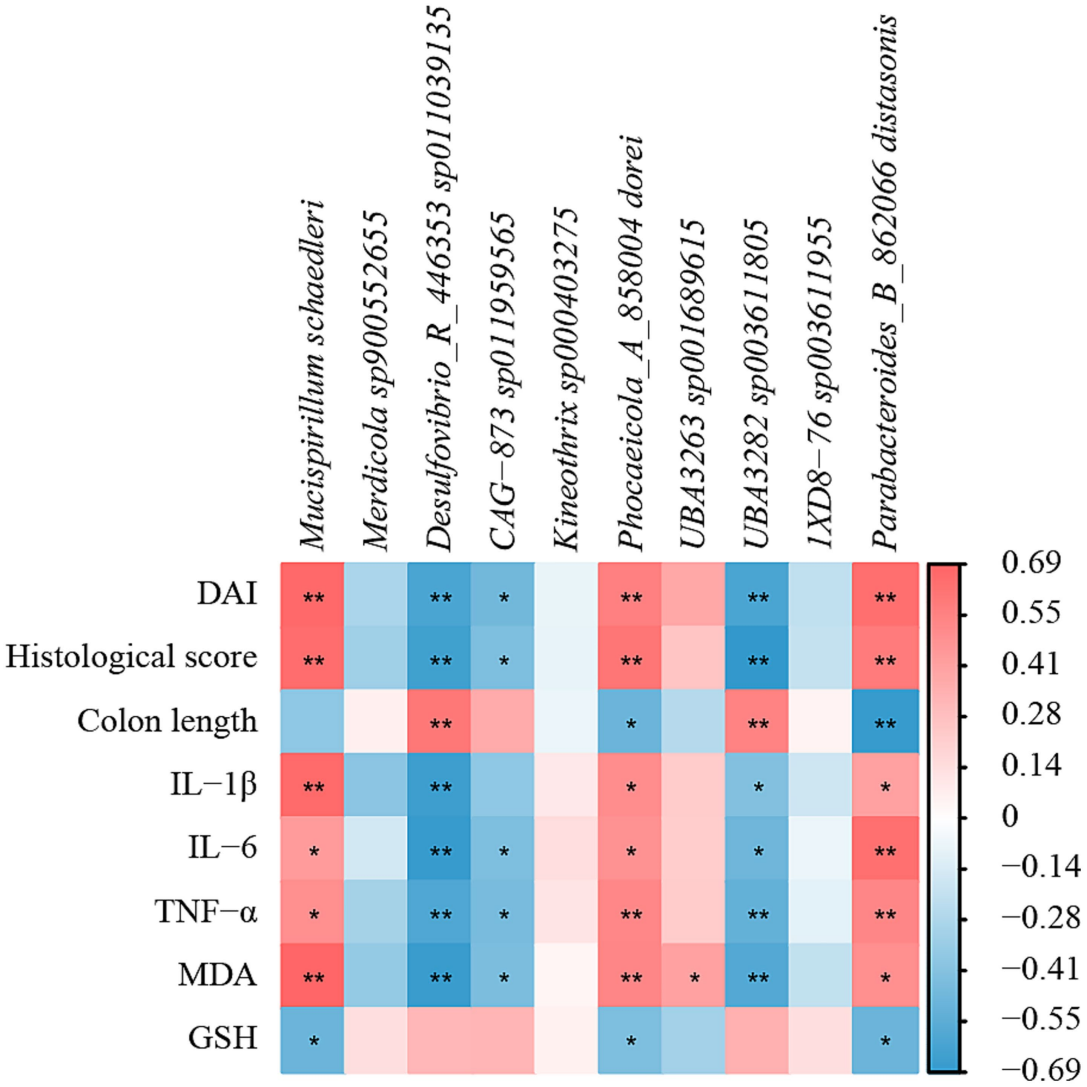
Correlation analysis of physicochemical parameters and intestinal flora of UC mice after AFE treatment. Heatmap of correlation between physicochemical parameters and intestinal flora. *p < 0.05, **p < 0.01.

Based on the Spearman algorithm, the correlation analysis between physiological and biochemical parameters and intestinal flora was performed (Figure 11). From the figure, it could be found that *Mucispirillum schaedleri*, *Phocaeicola_A_858004 dorei,* and *Parabacteroides_B_862066 distasonis* were positively correlated with DAI, histological scores, IL-1β, IL-6, TNF-α, and MDA, and negatively correlated with colon length and GSH, indicating that these species had a potential promoting effect on the formation of UC in mice. *Desulfovibrio_R_446353 sp011039135*, *CAG-873 sp011959565,* and *UBA3282 sp003611805* were negatively correlated with DAI, histological scores, IL-1β, IL-6, TNF-α, and MDA, but were positively correlated with colon length and GSH, and all were significant (*p* < 0.05), indicating that this part of the bacterial flora has a potential inhibitory effect on the formation of UC in mice.

## 4 Discussion

UC is a chronic disease-causing inflammation of the colon and rectum, affecting quality of life and increasing the risk of colorectal cancer. In the present study, the symptoms of diarrhea, bloody stools, weight loss, colon shortening, intestinal epithelial destruction, and intestinal barrier dysfunction were significantly improved in UC mice treated with AFE, indicating that AFE had the potential to be utilized as a pharmaceutical intervention for the management of UC.

**Figure 12:**
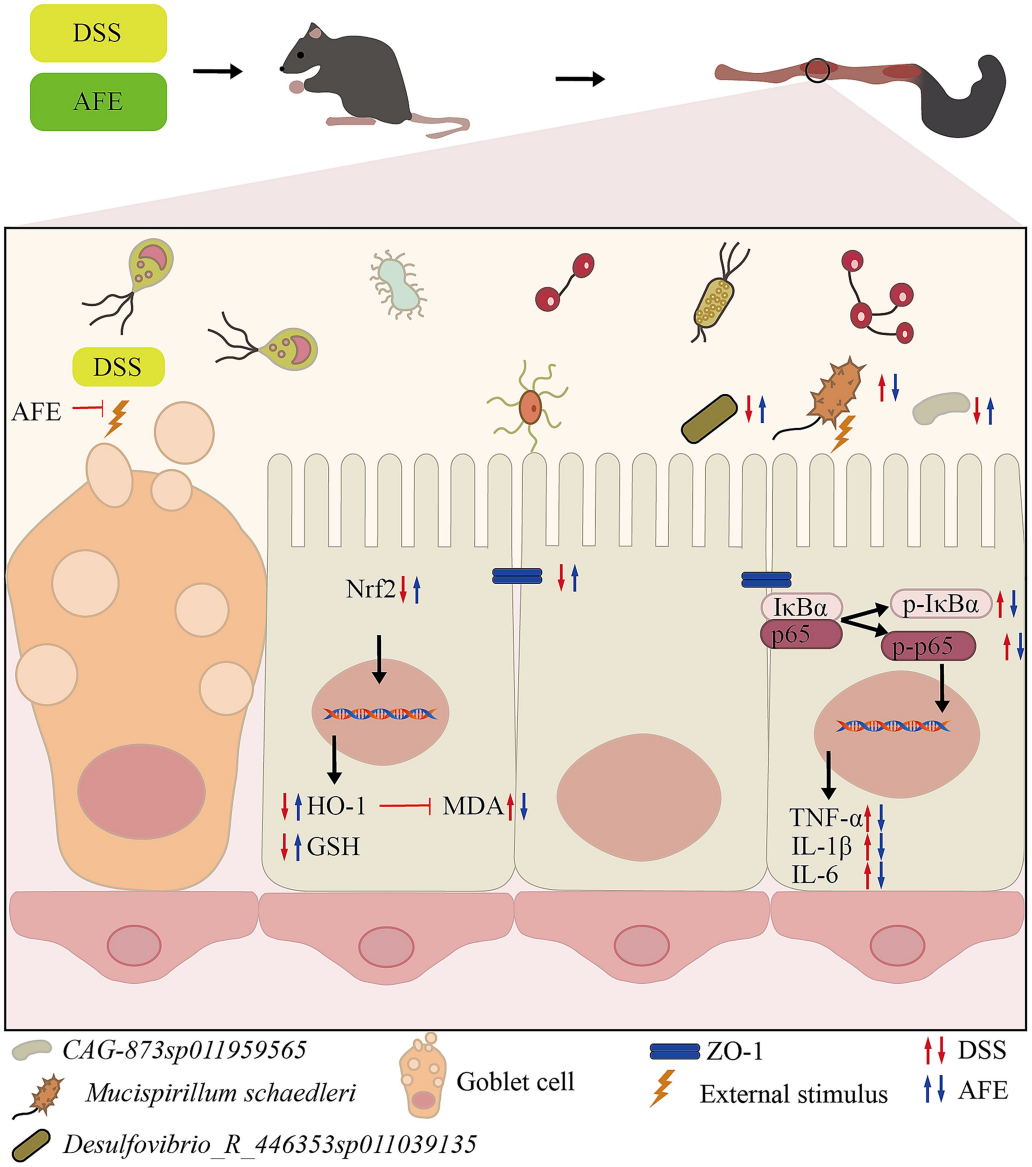
The mechanism of AFE alleviates DSS-induced UC.

Excessive intestinal inflammation is one of the main pathogenic factors of UC. Existing data show that in animal models of UC, there is obvious inflammation and immune dysfunction, which is mainly manifested in the imbalance of inflammatory factor secretion, such as TNF-α, IL-1β, and IL-6, significantly increased compared with normal levels (24). The continued increase in proinflammatory cytokines causes mucosal inflammation, leading to weakened colonic epithelial cell function (25). Therefore, maintaining the balance of inflammatory factors and restoring the body’s immune function to normal is crucial for the treatment of UC. Our results showed that the levels of TNF-α, IL-1β, and IL-6 in the colon tissue of UC mice were significantly increased, indicating that there was obvious inflammation, which was consistent with the previous reports. AFE treatment significantly reduced these levels, indicating that the therapeutic ability of AFE on UC is partly due to its anti-inflammatory function (Figure 12).

To explore the possible anti-inflammatory mechanism of AFE, our study detected key proteins of the NF-κB signaling pathway in colon tissue. The NF-κB signaling pathway plays an important role in the occurrence and development of inflammation and is closely related to the pathogenesis of UC (26). NF-κB is a heterodimer, usually composed of two subunits, p50 and p65. In its inactive state, NF-κB binds to IκBα in the cytoplasm. Once cells are stimulated by exogenous substances (such as lipopolysaccharide) or proinflammatory cytokines, IκBα is phosphorylated, free NF-κB is translocated to the cell nucleus, and induces the production of inflammatory factors (including TNF-α, IL-1β and IL-6), thereby promoting inflammation (27). The results showed that p-p65 and p-IκBα were significantly increased, and the NF-κB signaling pathway was significantly activated. These proteins were significantly reduced after AFE-H treatment, indicating that AFE-H could significantly inhibit the NF-κB signaling pathway, which is consistent with the previous inflammatory factor detection results. AFE alleviated DSS-induced inflammation in UC mice, which was associated with inhibition of the NF-κB signaling pathway (Figure 12).

Antioxidant therapy have great prospects in the treatment of UC. MDA and GSH were used to assess the level of oxidative stress (28). MDA is a product of intracellular lipid peroxidation, which can bind to proteins, nucleic acids, and other biomolecules in cells, leading to cell damage and inflammation (29). GSH is a tripeptide composed of glutamic acid, cysteine, and glycine. It is the most important antioxidant in cells and can remove free radicals and protect cells from oxidative stress (30). Our results showed that AFE-M and AFE-H treatment significantly reduced MDA and increased GSH in colon tissues of UC mice, which was associated with a mitigation of oxidative stress injury. This suggested a contribution from the antioxidant function of AFE to its overall therapeutic effect on UC (Figure 12).

In the body, the Nrf2/HO-1 signaling pathway is mainly responsible for regulating the expression of antioxidant proteins (31). The Nrf2/HO-1 signaling pathway is a classic pathway that regulates oxidative stress in cells. It helps cells remove excessive reactive oxygen species by upregulating the expression of a series of antioxidant enzymes, such as superoxide dismutase, catalase, and heme oxygenase 1 (HO-1), thereby protecting cells from oxidative damage (32). HO-1 is an effector protein downstream of the Nrf2/HO-1 signaling pathway that can partially decompose heme into biliverdin. Biliverdin and its product bilirubin are highly effective antioxidants (33). Our results showed that Nrf2 and HO-1 in the colon tissue of UC mice were reduced, indicating that the Nrf2/HO-1 signaling pathway was inhibited. Combined with previous antioxidant test results, AFE might alleviate oxidative stress damage in UC mice by activating the Nrf2/HO-1 signaling pathway (Figure 12).

Destruction of the intestinal mucosal barrier is a pathogenic factor of UC. Goblet cells are an important component of the intestinal mucosal barrier. The mucus proteins secreted by goblet cells can bind to invading pathogens, thereby preventing them from attacking host cells (34). Tight junction proteins (such as ZO-1, Occludin) are important components of the intestinal mucosal mechanical barrier and are the hallmark proteins of intestinal mucosal barrier integrity (35). During the development of UC, the expression level of tight junction proteins decreases, leading to increased intestinal mucosal permeability, which in turn produces a series of inflammatory reactions and clinical symptoms such as diarrhea and bloody stools (36). Our immunohistochemical results showed that AFE could reverse the decreased expression level of ZO-1 in the colon tissues of UC mice. In summary, AFE could protect the intestinal mucosa by increasing tight junction protein expression and safeguarding goblet cells.

Intestinal flora plays an important role in the formation and drug treatment of UC (37). Compared with healthy people, the diversity of intestinal flora in UC patients is reduced, and the composition varies greatly. The invasion of a large number of pathogenic microorganisms will trigger a strong intestinal immune response, leading to intestinal inflammation and digestive dysfunction (38). Therefore, maintaining intestinal flora homeostasis is crucial for the treatment of UC.

In order to explore the regulatory effect of AFE on intestinal flora, we analyzed the structure of mouse intestinal flora at the phylum level. Many studies have shown that the intestinal flora structure in UC patients or animal models is significantly disordered, and the diversity and richness of intestinal flora are reduced, mainly manifested in the increase in the relative abundance of harmful bacteria, such as *Proteobacteria* (39), D*eferribacterota* (40), *Bacteroidota* (41), and the decrease in the relative abundance of beneficial bacteria, such as *Desulfobacterota_I* (42), *Firmicutes* (43). A similar situation was observed in UC mice in our study (Figure 9). However, AFE treatment reversed the decreased diversity and richness of the intestinal flora in UC mice at the phylum level, reducing the relative abundance of pathogenic bacteria and increasing the relative abundance of beneficial bacteria, indicating that the therapeutic effect of AFE on UC mice was partly attributed to the regulatory effect on the intestinal flora.

Our correlation analysis showed that the changes in the relative abundance of *Mucispirillum schaedleri*, *Desulfovibrio_R_446353 sp011039135,* and *CAG-873 sp011959565* were closely associated with the onset of UC (Figure 12). *Mucispirillum schaedleri* belongs to the *Deferribacterota*, is commonly found in the intestinal microbiota of rodents, pigs, and humans, and is closely related to intestinal inflammation (44, 45). On the one hand, *Mucispirillum schaedleri* can protect against UC caused by *Salmonella typhimurium* by inhibiting pathogen invasion (45). On the other hand, *Mucispirillum schaedleri* also has a high pathogenic potential, being invasive enough to trigger an adaptive immune response in healthy hosts or an intestinal inflammatory response in severely immunocompromised hosts, and accumulating significantly in inflamed colonic tissue (44). Our results showed that the relative abundance of *Mucispirillum schaedleri* increased significantly in UC mice, indicating pathogenicity. After AFE treatment, the relative abundance of *Mucispirillum schaedleri* decreased, suggesting that AFE has a positive effect on the intestinal flora of UC mice. *Desulfovibrio_R_446353sp011039135* is a member of the *Desulfovibrio. Desulfovibrio* is the main producer of hydrogen sulfide in the gastrointestinal tract. Its production of hydrogen sulfide has a certain regulatory effect on intestinal inflammation (46). One study found that the relative abundance of *Desulfovibrio* was significantly reduced in DSS-induced UC mice, which was restored after treatment (47). Our results are similar to that;, therefore, *Desulfovibrio_R_446353sp011039135* may be a potentially beneficial bacterium. There are a few related reports on *CAG-873sp011959565*. Combined with our results, we believe that this strain is a potentially beneficial bacterium. In addition, the intestinal flora of UC mice was disordered not only in structure and composition but also in function, and AFE treatment could significantly reverse these abnormal changes, indicating that AFE can regulate the intestinal flora in multiple ways.

Among the active ingredients of AFE, naringin, neohesperidin, and naringin rutaecarpon account for a large proportion. Naringin, neohesperidin, and narirutin account for a large proportion of the active ingredients in AFE and may be the main active ingredients. Existing data show that naringin can reduce inflammation, oxidative stress, and apoptosis of colonic epithelial cells caused by UC, and can also reduce damage caused by apoptosis and inflammation in human umbilical vein endothelial cells, downregulate the expression of IL-1β, IL-6, and IL-18, and has a good anti-inflammatory effect (48, 49). Neohesperidin can reduce myocardial damage, oxidative stress, apoptosis, and immune imbalance by inhibiting the phosphorylation of p65, and can also reduce obesity by changing the composition of the intestinal microbiota of mice fed a high-fat diet (50, 51). Narirutin treated DSS-induced UC mice by regulating intestinal flora (8). For other compounds, hesperidin treats DSS-induced UC by maintaining the colonic epithelial barrier by blocking the RIPK3/MLKL signaling pathway (52). Nobiletin improves hepatic lipid deposition, oxidative stress, and inflammation in non-alcoholic fatty liver disease via the Nrf2/NF-κB signaling pathway (53). These compounds may exhibit multiple mechanisms in treating UC, and the next step is to test the combination of these compounds in UC mice.

The present study demonstrated that AFE effectively ameliorates UC, as evidenced by improved epithelial integrity and reduced inflammation. However, a key question arising from our findings was whether these protective effects were primarily due to the direct action of AFE constituents on specific cell types (e.g., epithelial cells, immune cells), or secondary to its potent anti-inflammatory activity. For instance, certain components in AFE might activate EGFR pathways, which are crucial for epithelial proliferation and migration. Alternatively, the improved epithelial physiology might be largely secondary to the resolution of inflammation. By suppressing pro-inflammatory cytokines (e.g., TNF-α, IL-6, IL-1β) via modulation of immune cells like macrophages, AFE created a microenvironment conducive to intrinsic healing processes. In this scenario, certain components’ primary target might be immune cells. It was most likely that both direct and indirect mechanisms contribute to the overall efficacy of AFE. Dissecting the primary versus secondary effects represents a major focus for our future research. Approaches will include *in vitro* co-culture systems of epithelial and immune cells, and isolation of specific components to identify the key active compounds responsible for each observed effect.

Interestingly, our results showed that AFE-H was more effective than SASP, which may be due to the relatively low dose of SASP we selected in our experiments. Unfortunately, we did not set up corresponding positive drugs when detecting the NF-κB signaling pathway and the Nrf2/HO-1 signaling pathway. In the next experiment, agonists or inhibitors of the corresponding pathways should be set up to further verify that AFE treats UC by stimulating or inhibiting the corresponding pathways.

## 5 Conclusion

We used HPLC to detect the effective parts in AFE, and then evaluated the therapeutic effect of AFE in treating UC mice. AFE has been demonstrated to alleviate symptoms such as bloody stools, diarrhea, and weight loss in murine models of UC. It had also been shown to ameliorate colon pathological damage, enhance intestinal mucosal barrier function, and possess anti-inflammatory and antioxidant properties. Its anti-inflammatory and antioxidant mechanisms were related to the inhibition of the NF-κB signaling pathway and the activation of the Nrf2 signaling pathway. AFE treatment was associated with a regulated composition of intestinal flora in UC mice, characterized by a relative suppression of harmful bacteria and an expansion of beneficial bacteria. These shifts suggest a restoration towards a more functionally normal microbial community.

## Data Availability

The data presented in the study are deposited in the NCBI repository, https://www.ncbi.nlm.nih.gov/, accession no. PRJNA1286950.
