# Supplementary figures and images for: Aurantii Fructus extract alleviates DSS-induced colitis in mice via regulating NF-κB and Nrf2/HO-1 signaling pathways and modulating intestinal microbiota
Source: Front Nutr. 2025 Oct 16;12:1661040. doi: 10.3389/fnut.2025.1661040 (PMC12571614; doi:10.3389/fnut.2025.1661040)

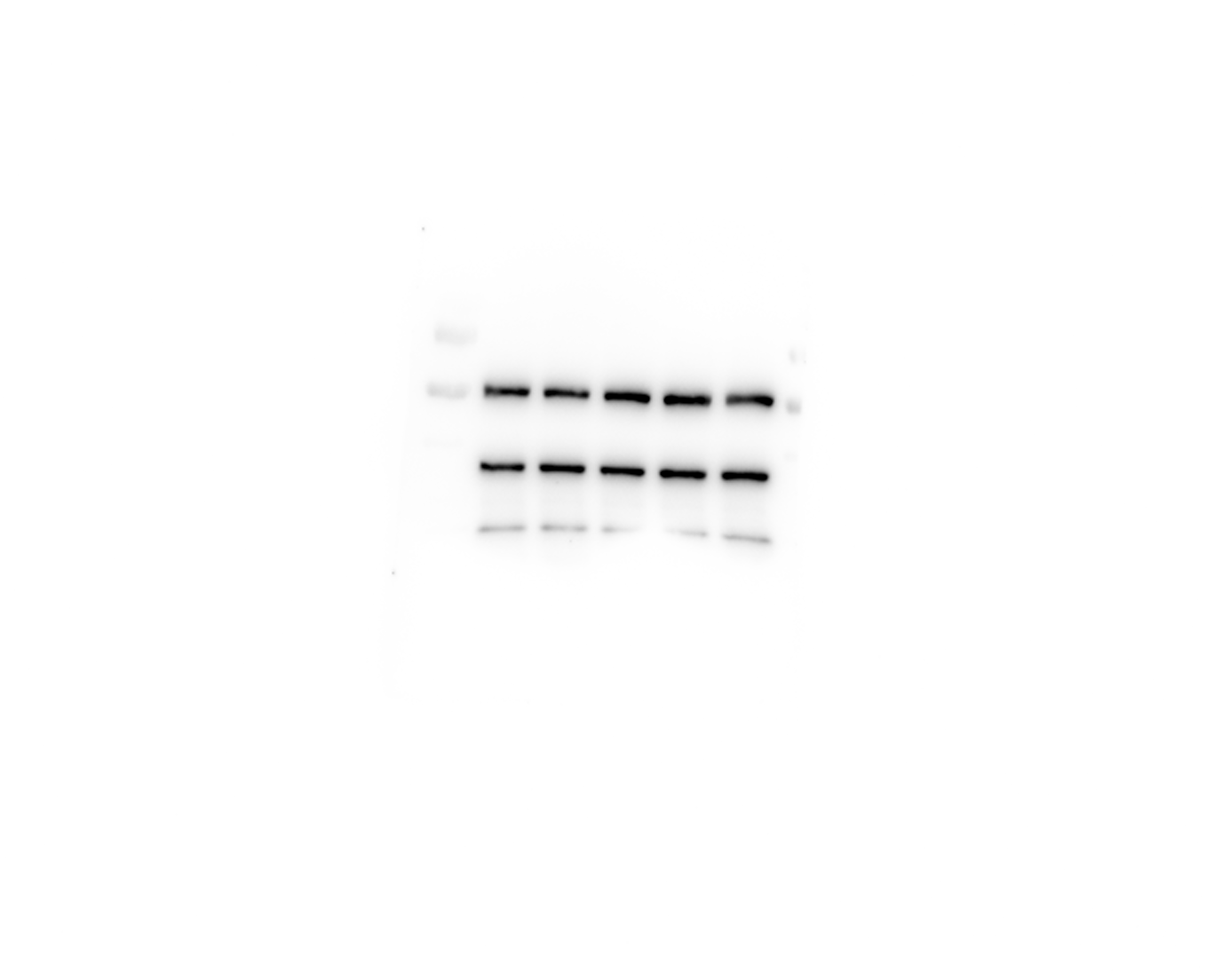

Supplement: Supplementary file 1 [file Data_Sheet_1.zip › wb original images of NF-a╩B/Ia╩Ba┴/Ia╩Ba┴ and GAPDH(1).tif]

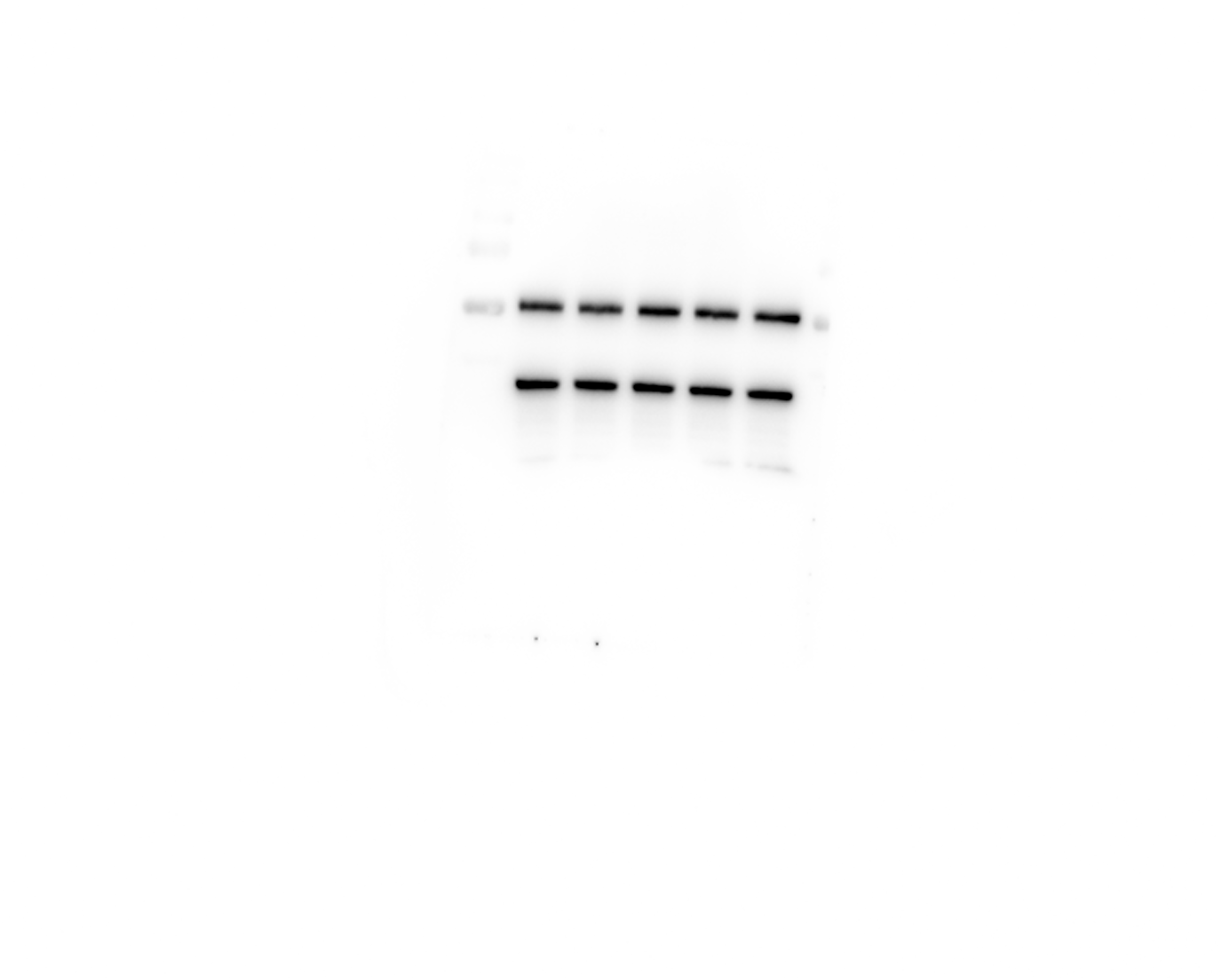

Supplement: Supplementary file 1 [file Data_Sheet_1.zip › wb original images of NF-a╩B/Ia╩Ba┴/Ia╩Ba┴ and GAPDH(2).tif]

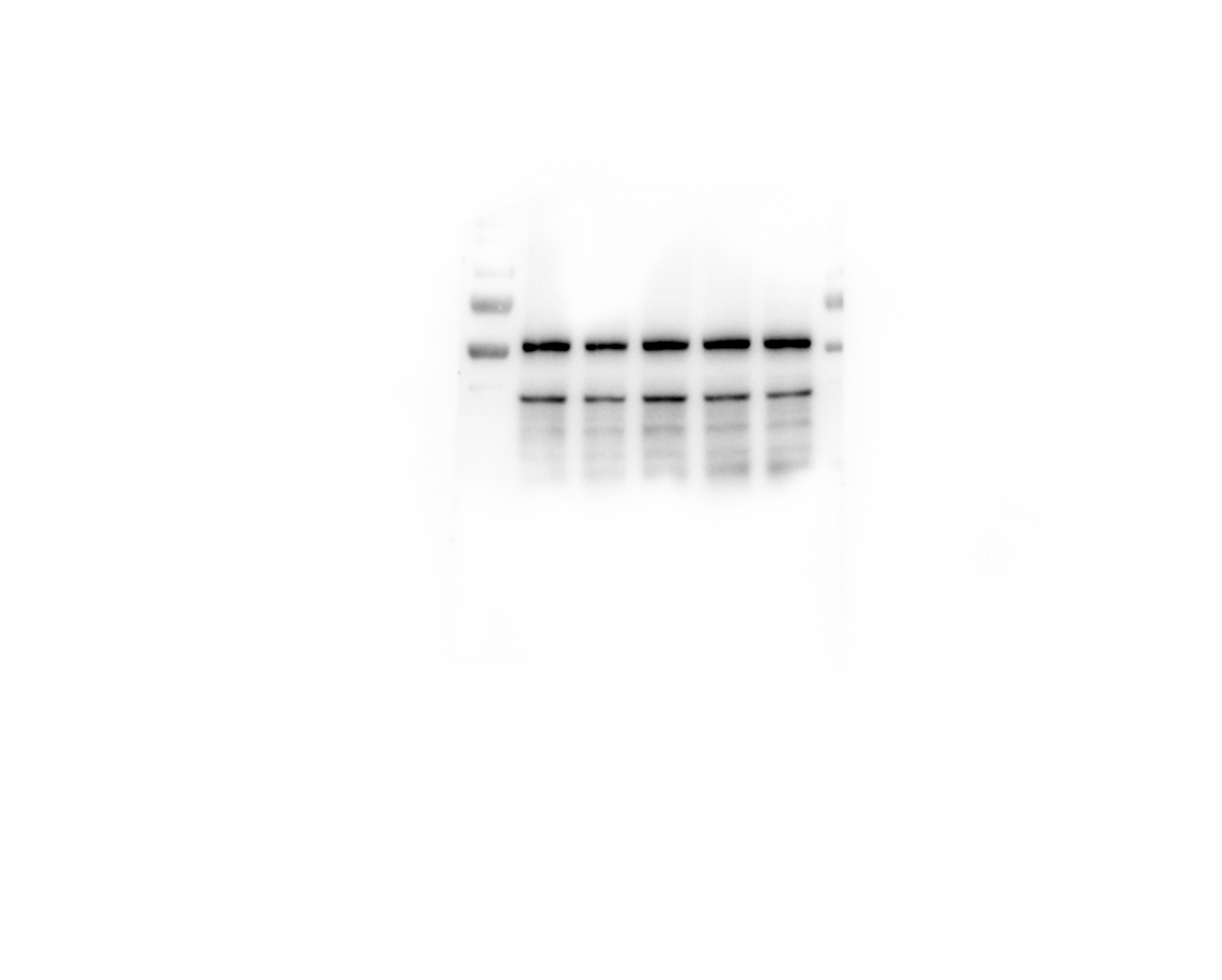

Supplement: Supplementary file 1 [file Data_Sheet_1.zip › wb original images of NF-a╩B/Ia╩Ba┴/Ia╩Ba┴ and GAPDH(3).tif]

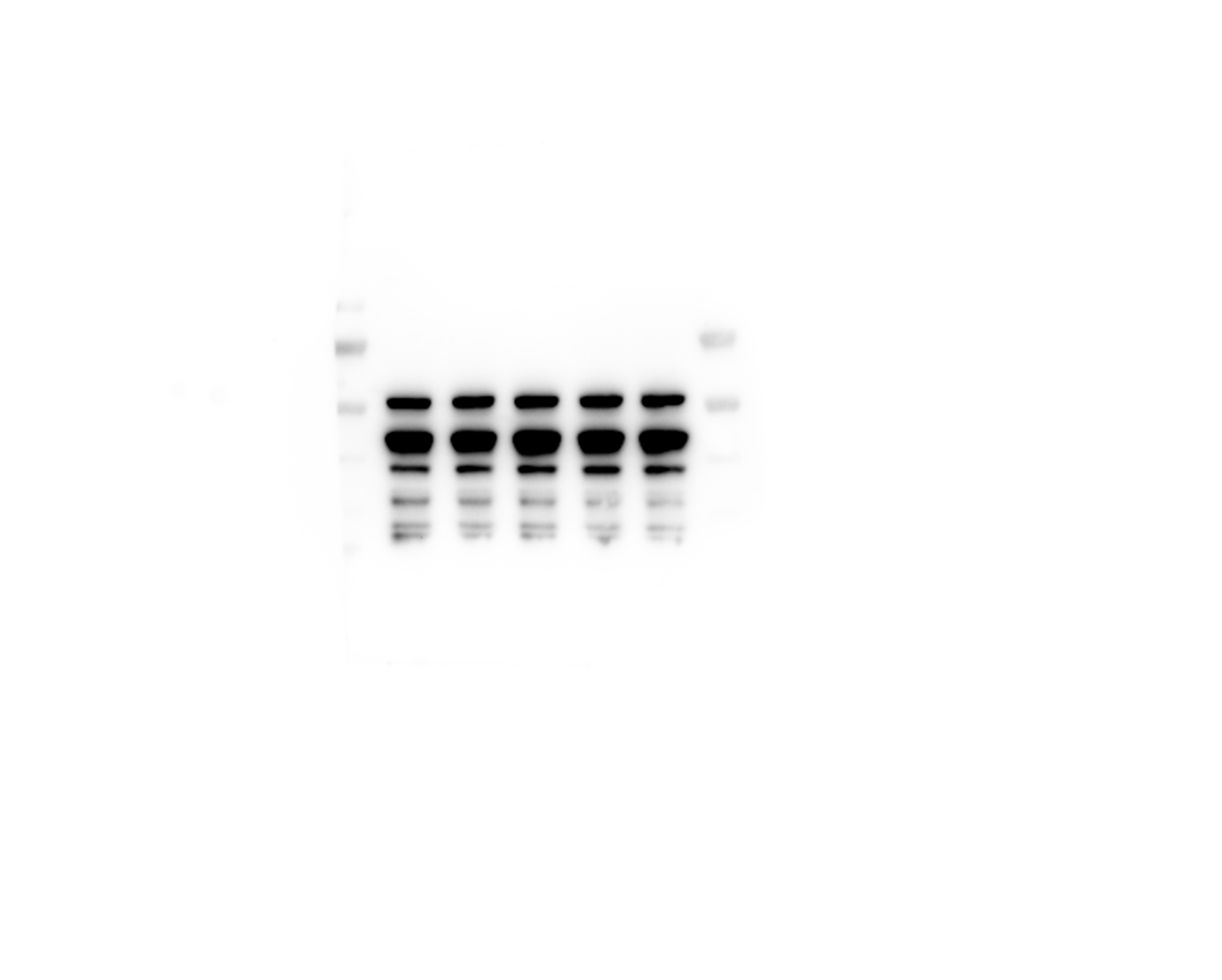

Supplement: Supplementary file 1 [file Data_Sheet_1.zip › wb original images of NF-a╩B/p65/p65 and a┴-tubulin(1).tif]

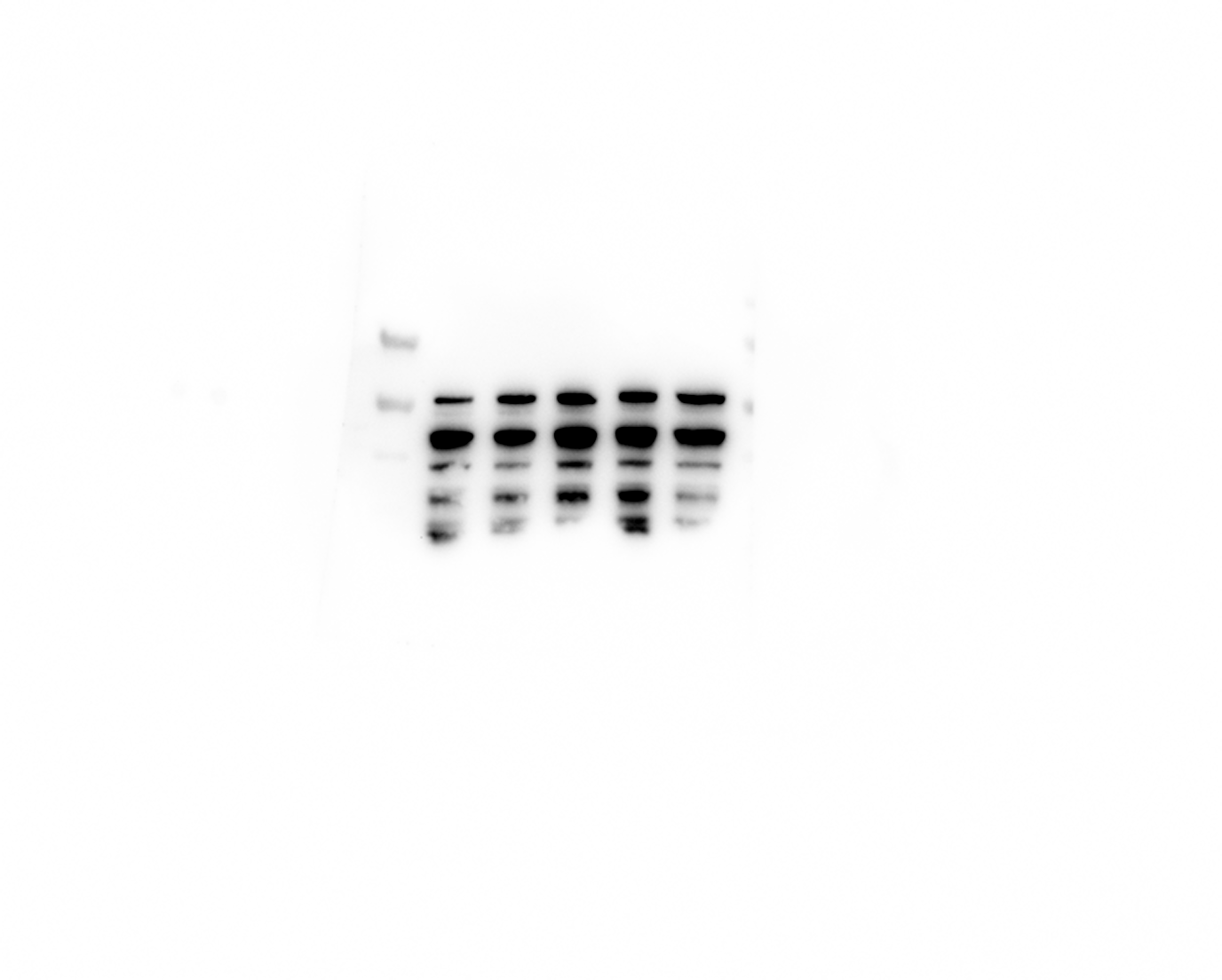

Supplement: Supplementary file 1 [file Data_Sheet_1.zip › wb original images of NF-a╩B/p65/p65 and a┴-tubulin(2).tif]

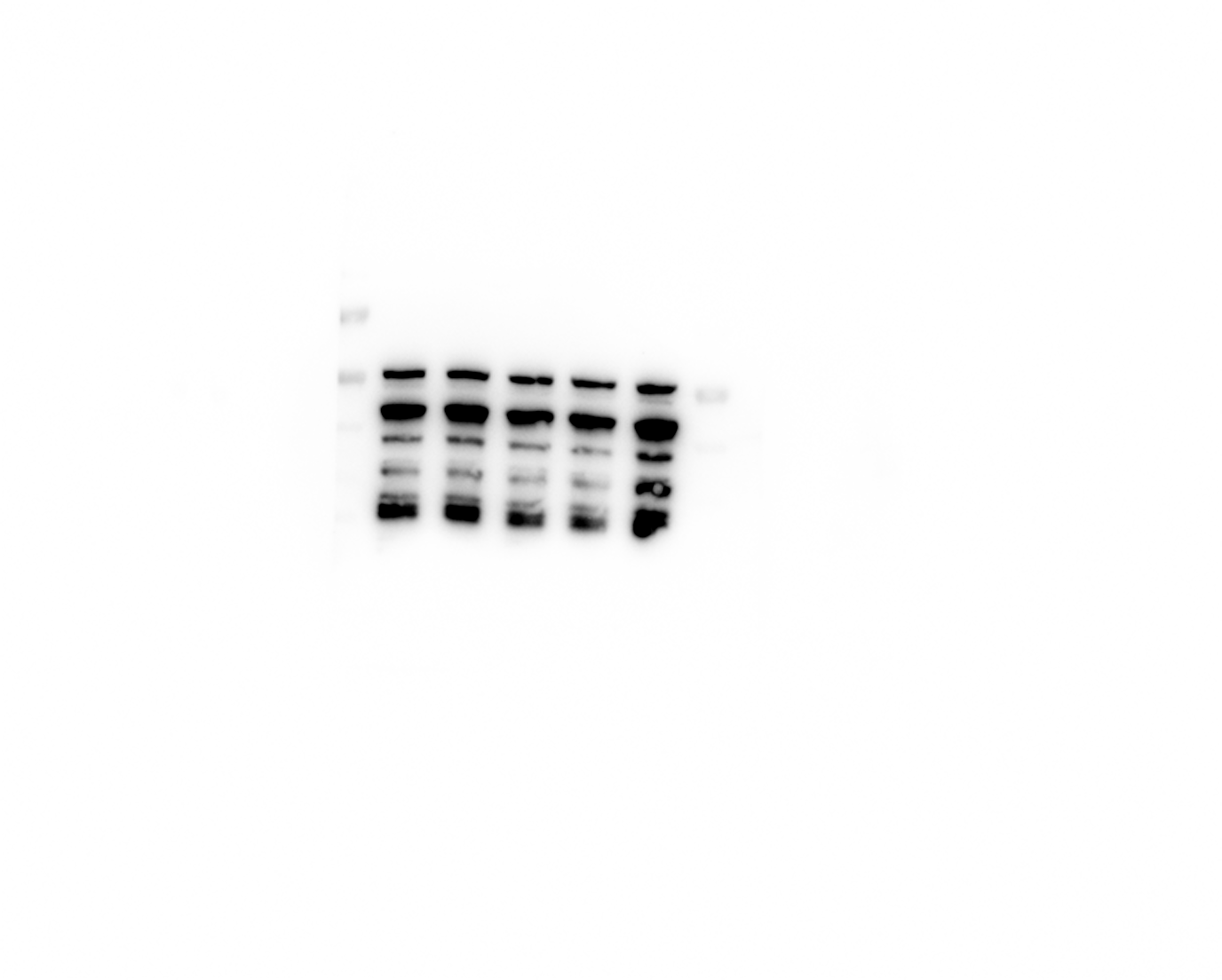

Supplement: Supplementary file 1 [file Data_Sheet_1.zip › wb original images of NF-a╩B/p65/p65 and a┴-tubulin(3).tif]

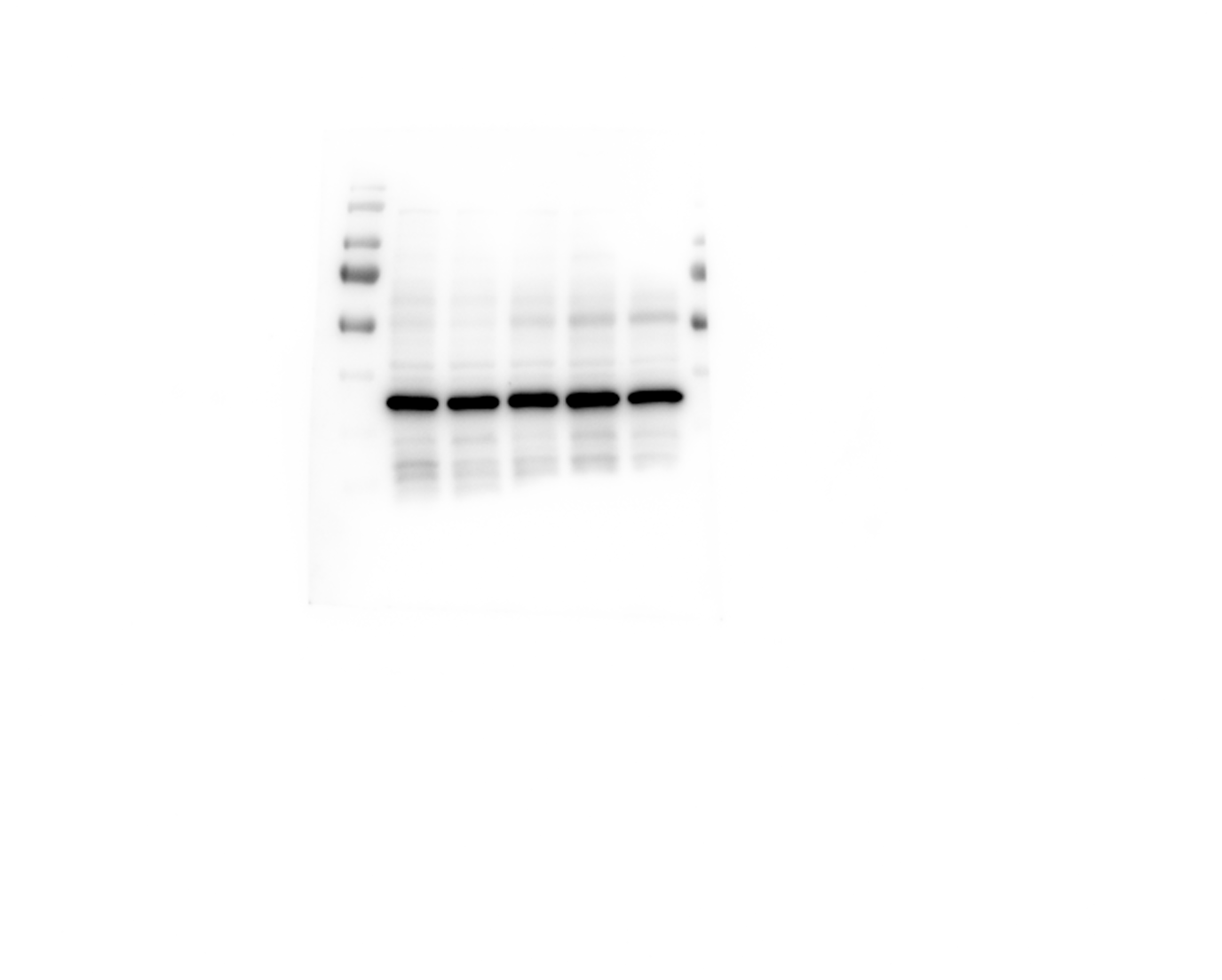

Supplement: Supplementary file 1 [file Data_Sheet_1.zip › wb original images of NF-a╩B/p-Ia╩Ba┴/1/GAPDH(1).tif]

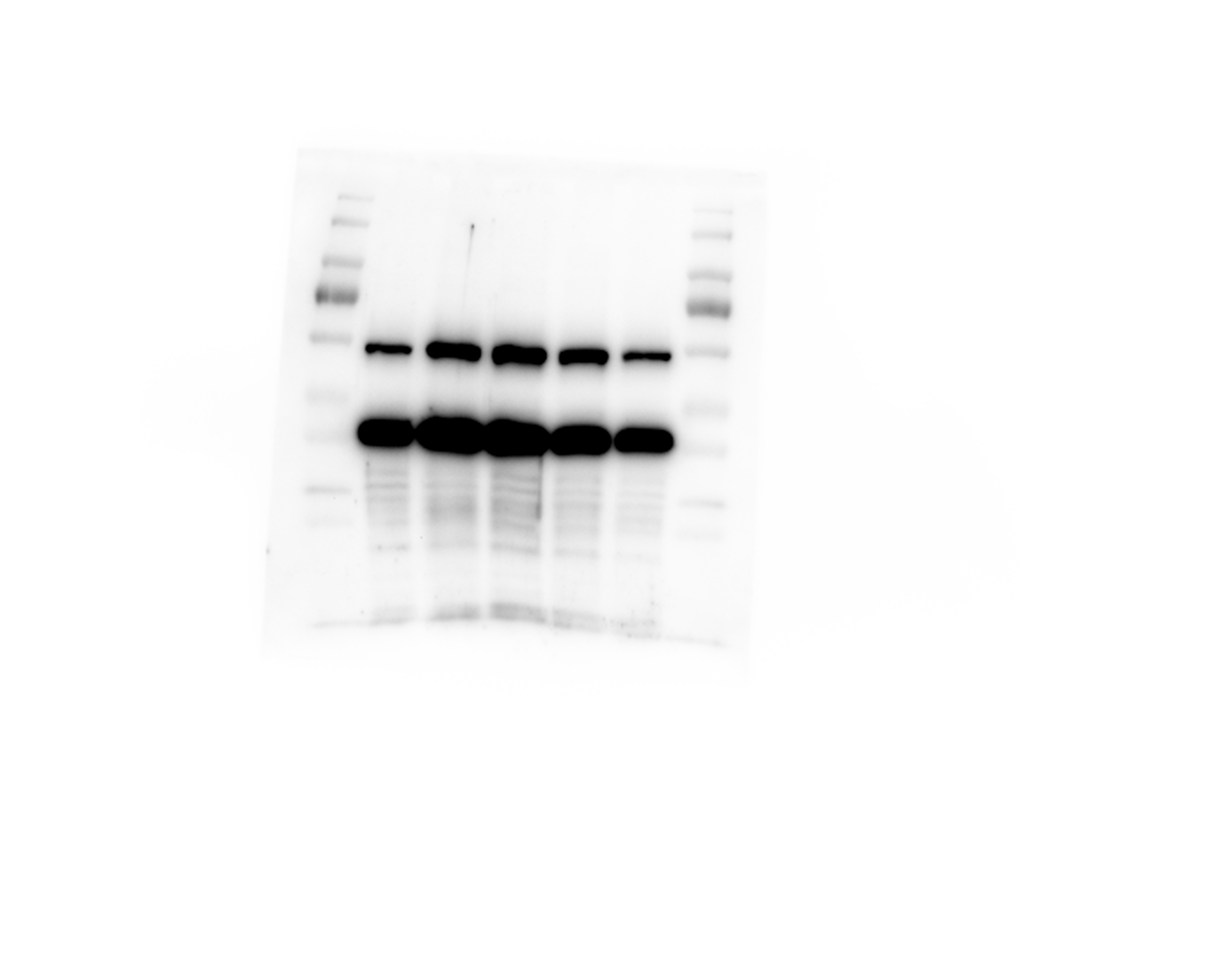

Supplement: Supplementary file 1 [file Data_Sheet_1.zip › wb original images of NF-a╩B/p-Ia╩Ba┴/1/p-Ia╩Ba┴(1).tif]

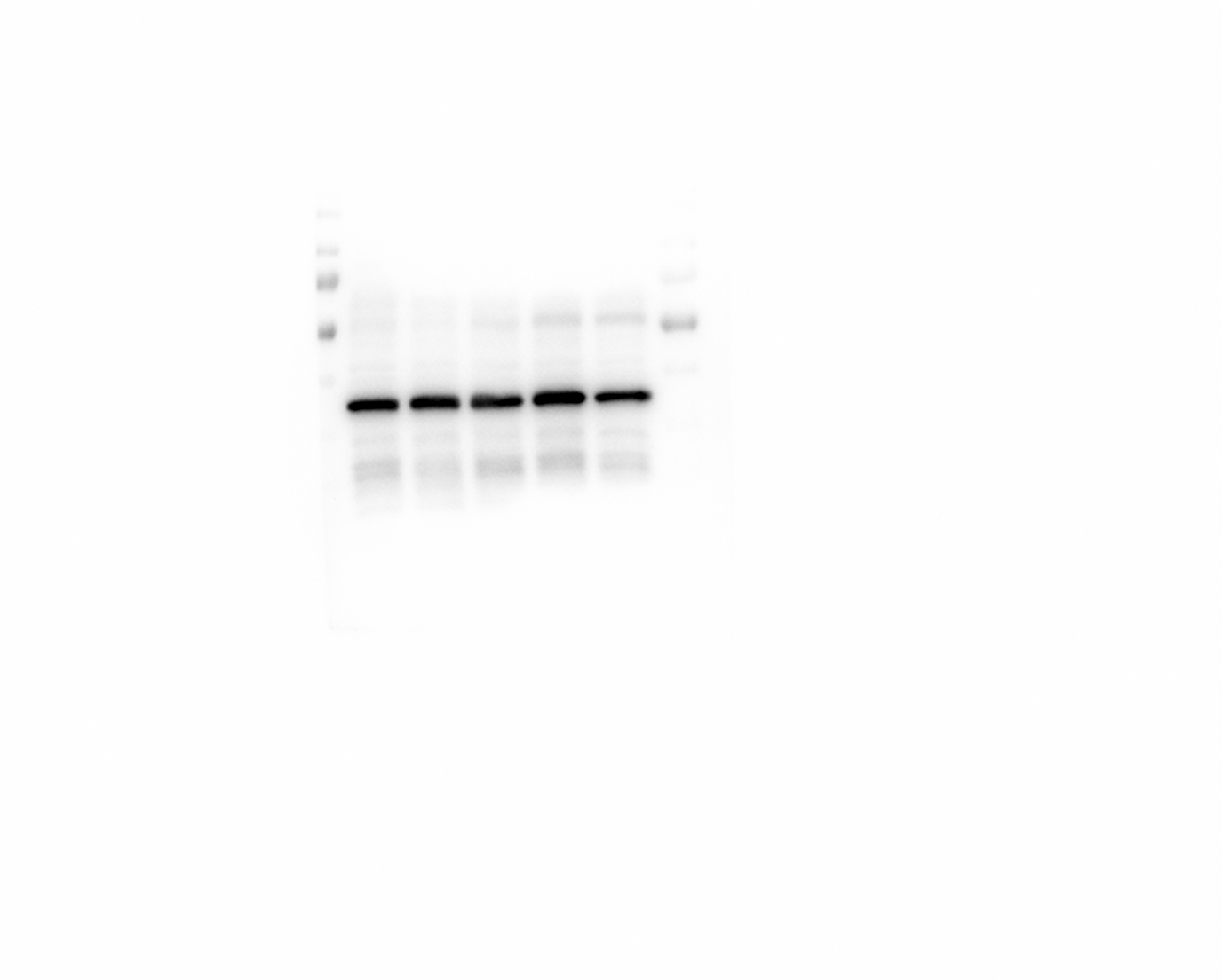

Supplement: Supplementary file 1 [file Data_Sheet_1.zip › wb original images of NF-a╩B/p-Ia╩Ba┴/2/GAPDH(2).tif]

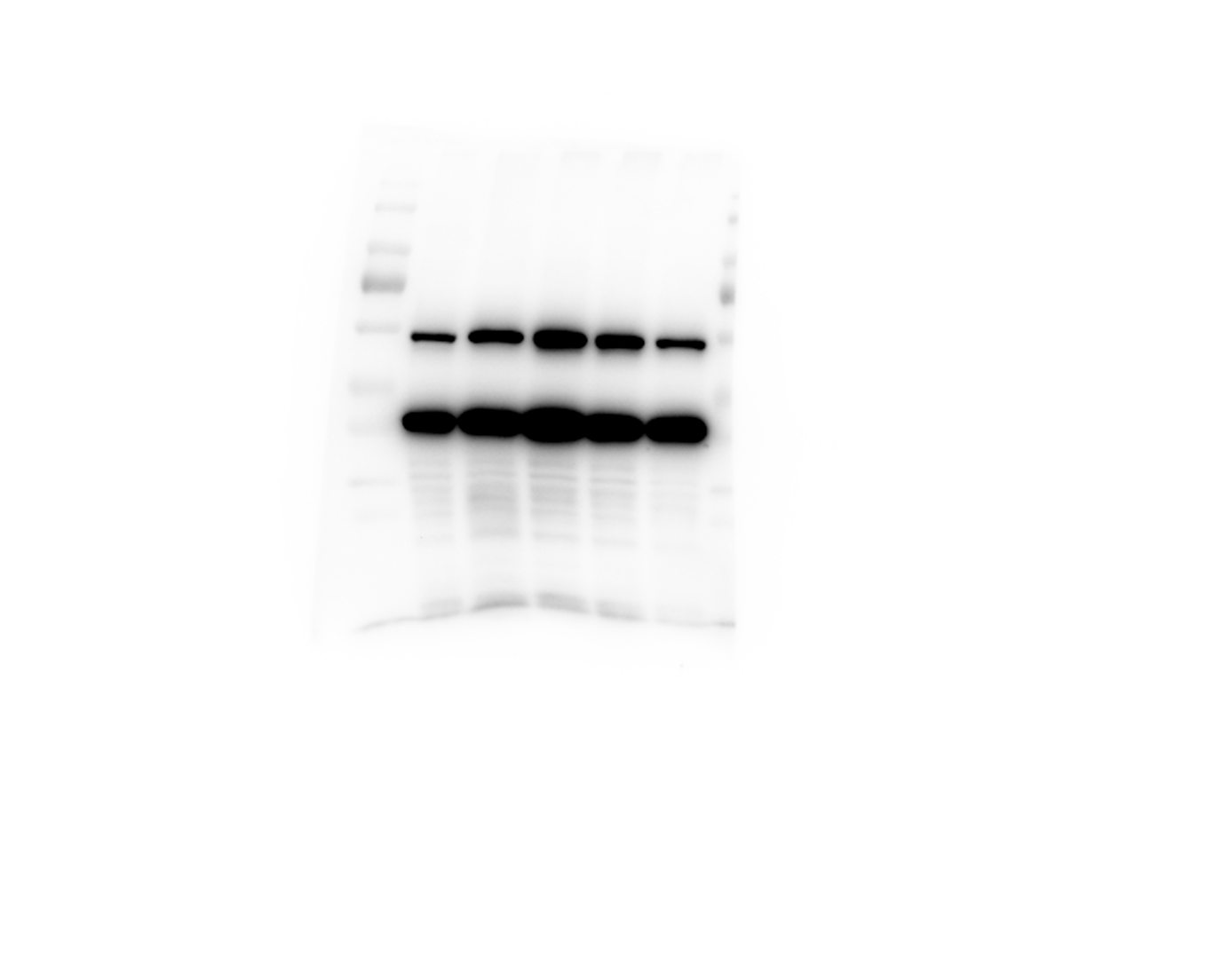

Supplement: Supplementary file 1 [file Data_Sheet_1.zip › wb original images of NF-a╩B/p-Ia╩Ba┴/2/p-Ia╩Ba┴(2).tif]

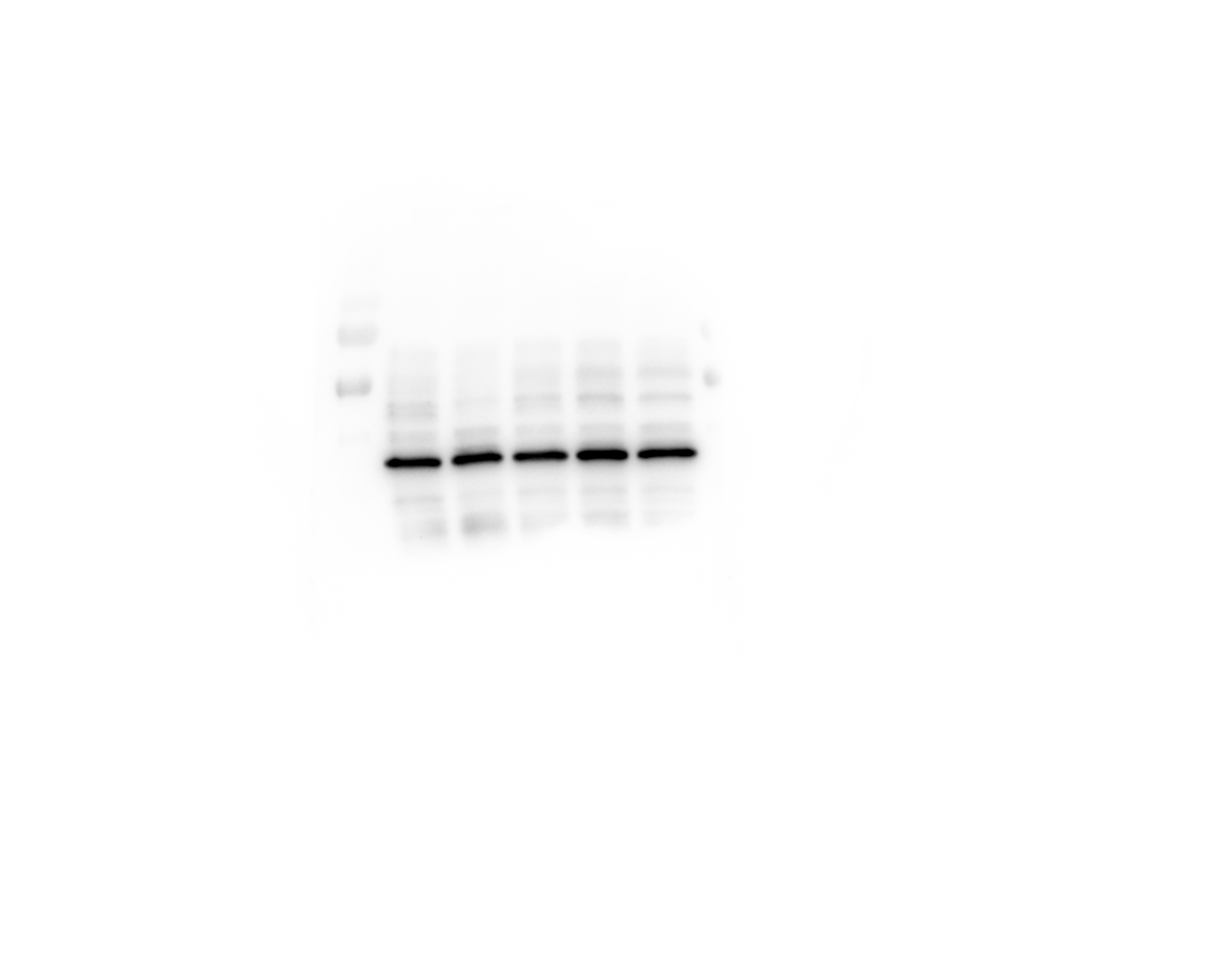

Supplement: Supplementary file 1 [file Data_Sheet_1.zip › wb original images of NF-a╩B/p-Ia╩Ba┴/3/GAPDH(3).tif]

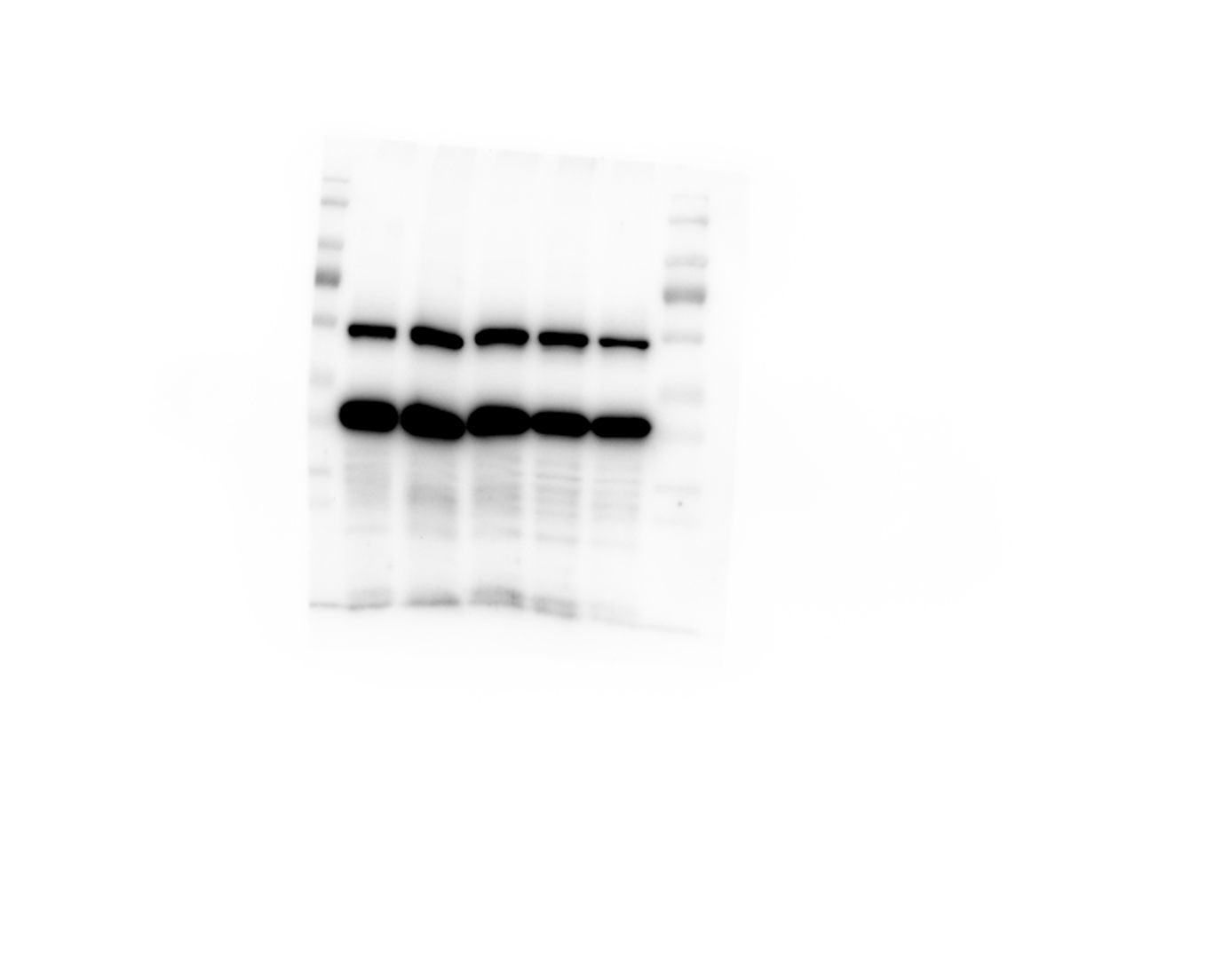

Supplement: Supplementary file 1 [file Data_Sheet_1.zip › wb original images of NF-a╩B/p-Ia╩Ba┴/3/p-Ia╩Ba┴(3).tif]

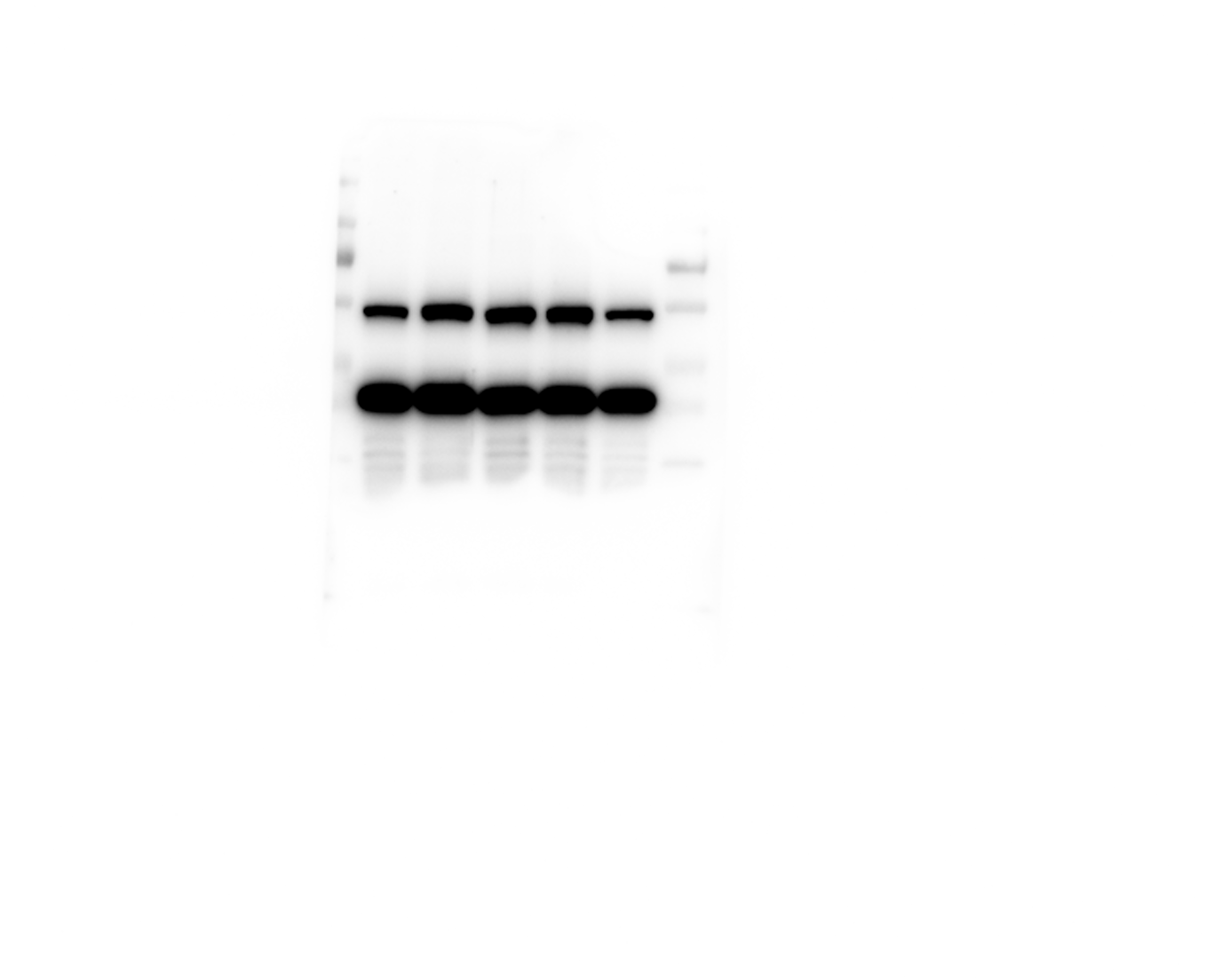

Supplement: Supplementary file 1 [file Data_Sheet_1.zip › wb original images of NF-a╩B/p-p65/p-p65 and a┴-tubulin(1).tif]

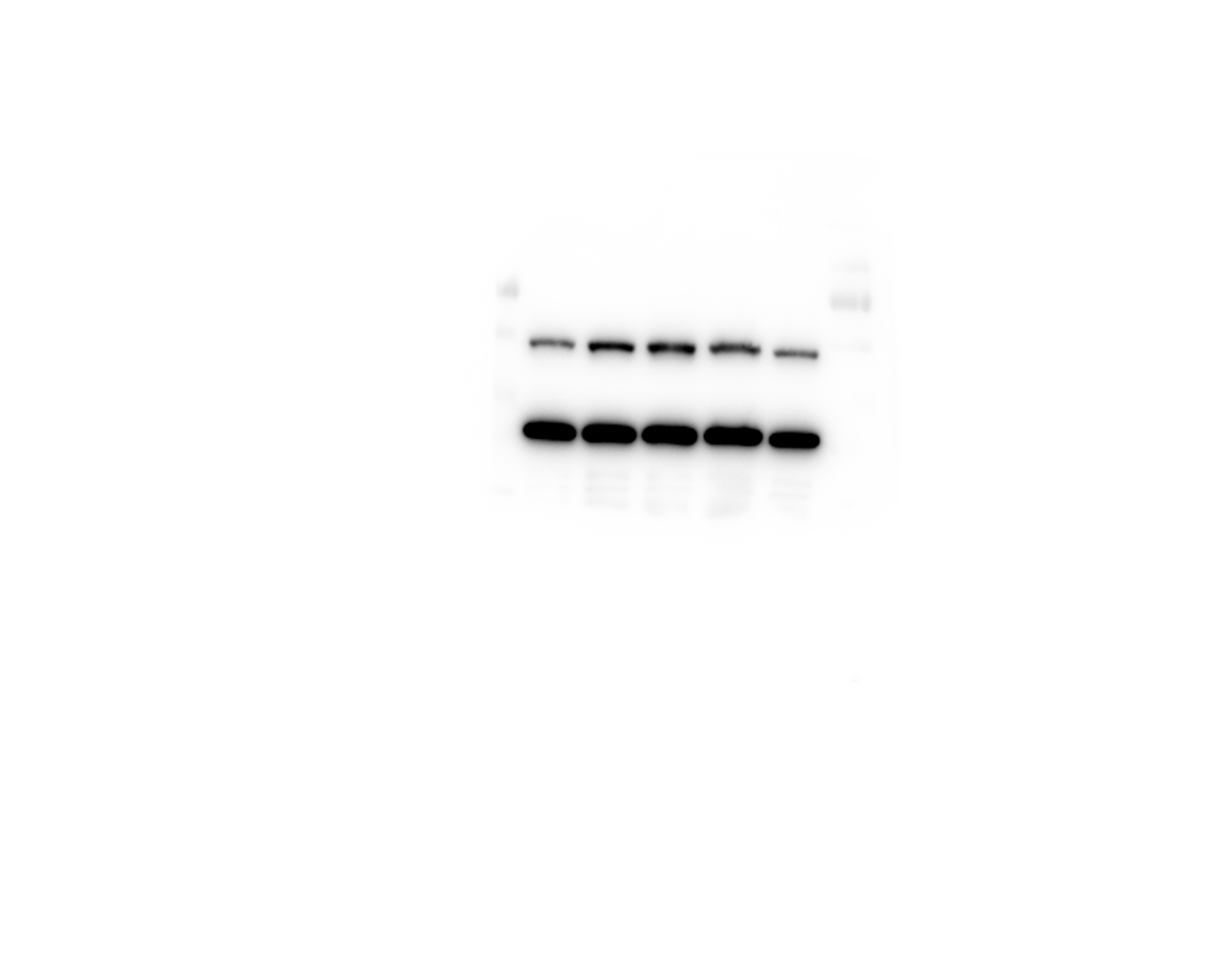

Supplement: Supplementary file 1 [file Data_Sheet_1.zip › wb original images of NF-a╩B/p-p65/p-p65 and a┴-tubulin(2).tif]

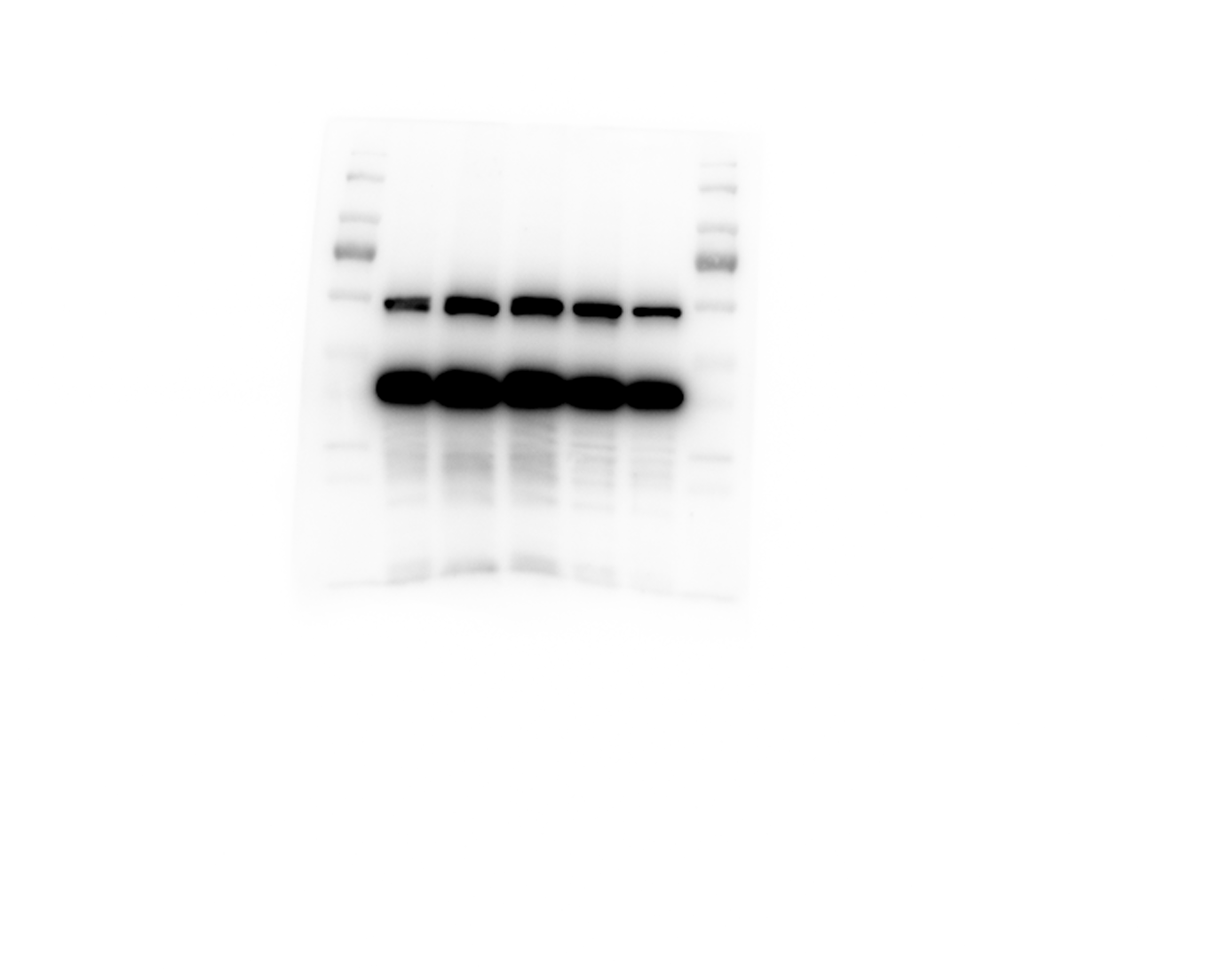

Supplement: Supplementary file 1 [file Data_Sheet_1.zip › wb original images of NF-a╩B/p-p65/p-p65 and a┴-tubulin(3).tif]
